# Amphiphilic Nanogels as Versatile Stabilizers for Pickering Emulsions

**DOI:** 10.1021/acsnano.4c05143

**Published:** 2024-09-04

**Authors:** Ruiguang Cui, Maret Ickler, Ante Markovina, Sidra Kanwal, Nicolas Vogel, Daniel Klinger

**Affiliations:** † Institute of Pharmacy, Freie Universität Berlin, Königin-Luise-Str. 2-4, Berlin 14197, Germany; ‡ Institute of Particle Technology, Friedrich-Alexander-Universität Erlangen-Nürnberg, Erlangen 91058, Germany

**Keywords:** nano/microgels, Pickering emulsions, phase inversion, Flory−Huggins parameter, interfacial tension

## Abstract

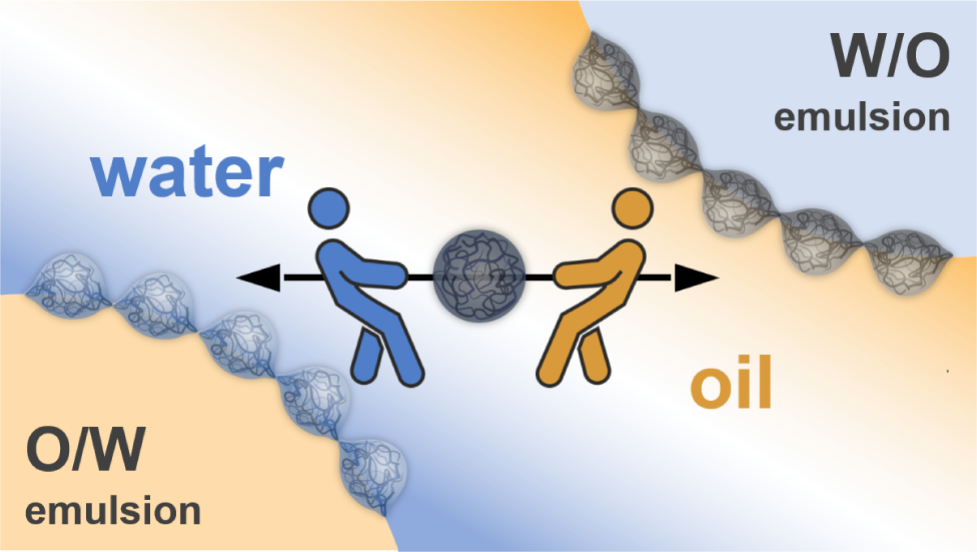

Pickering emulsions (PEs) are stabilized by particles at the water/oil interface and exhibit superior long-term stability compared to emulsions with molecular surfactants. Among colloidal stabilizers, nano/microgels facilitate emulsification and can introduce stimuli responsiveness. While increasing their hydrophobicity is connected to phase inversion from oil-in-water (O/W) to water-in-oil (W/O) emulsions, a predictive model to relate this phase inversion to the molecular structure of the nano/microgel network remains missing. Addressing this challenge, we developed a library of amphiphilic nanogels (ANGs) that enable adjusting their hydrophobicity while maintaining similar colloidal structures. This enabled us to systematically investigate the influence of network hydrophobicity on emulsion stabilization. We found that W/O emulsions are preferred with increasing ANG hydrophobicity, oil polarity, and oil/water ratio. For nonpolar oils, increasing emulsification temperature enabled the formation of W/O PEs that are metastable at room temperature. We connected this behavior to interfacial ANG adsorption kinetics and quantified ANG deformation and swelling in both phases via atomic force microscopy. Importantly, we developed a quantitative method to predict phase inversion by the difference in Flory–Huggins parameters between ANGs with water and oil (χ_water_ – χ_oil_). Overall, this study provides crucial structure–property relations to assist the design of nano/microgels for advanced PEs.

## Introduction

Pickering emulsions (PEs) show excellent long-term stability due to their stabilization by nano- or microparticles at the oil/water interface.^1,2^ As a result, PEs often outperform surfactant-stabilized emulsions in applications such as food processing,^3−5^ pharmaceutics,^6−9^ cosmetics,^10−12^ catalysis,^13−18^ and biomimetic microreactors.^19^

Traditionally, solid particles are used in PEs, but polymer nano/microgels have emerged as softer colloidal alternatives with distinct advantages.^20−22^ For example, the effective interfacial adsorption of these highly swollen particles requires a much lower energy input for emulsification.^23,24^ In addition, a stimuli-responsive swelling or degradation of the colloidal stabilizers can impart advanced properties into PEs, such as triggered destabilization^25−29^ or phase inversion.^30,31^ However, precisely controlling and predicting such features based on the nano/microgel structure remains challenging due to the complex behavior of these soft colloids at the oil/water interface. While solid particles show a well-defined contact angle that indicates their wettability and determines the type of emulsion (oil-in-water O/W or water-in oil W/O),^32−34^ nano/microgels deform at the interface.^35−41^ This deformation prevents the use of contact angles as an indicator to predict typical emulsion properties such as emulsion type, stability, and droplet size. Consequently, the underlying droplet stabilization is more complex and coupled to the interplay between interfacial swelling, mechanical deformability, adsorption kinetics, and interfacial tension.

Up to now, it is established that oil-in-water (O/W) emulsions are stabilized by hydrophilic nano/microgels and their preferential interaction with the water phase (Finkle’s rule).^42^ In contrast, water-in-oil emulsions (W/O) require a strong interaction of the nano/microgel networks with the oil phase.^43,44^ Hydrophilic nano/microgels can achieve this through hydrogen bonding between the network polymer and specific oil molecules, e.g., hexanol,^45^ octanol,^45,46^ and other fatty alcohols.^27^ Here, the nano/microgels are still located preferably in the water phase and stabilization occurs through cohesive multilayer arrangement of nano/microgels.^45^ Stabilization of water droplets in other oils requires more hydrophobic nano/microgels, e.g., composites from hydrophilic microgels with hydrophobic polymers or inorganic materials.^27,47^ In such cases, favored microgel-oil interactions can enhance protrusion of the particles into the oil phase, thus stabilizing the W/O system.^47^ In addition, the interaction of hydrophobic particles from the oil phase with hydrophilic particles from the water phase can form particle complexes at the oil/water interface that are able to stabilize W/O emulsions.^46,48,49^

While these existing reports provide coarse guidance to select nano/microgels for a specific emulsion type, a quantitative approach to correlate the molecular network structure with the emulsion properties does not exist. To address these shortcomings, the swelling of nano/microgels in water and oil was proposed as a more quantitative approach recently.^43^ While this concept draws analogies to the contact angle for solid nanoparticles, the connection between nano/microgel hydrophobicity, interfacial behavior, and the ability to stabilize emulsions is still not fully understood. Thus, the accurate design of nano/microgels to program specific emulsion properties (e.g., W/O or O/W) requires a quantitative approach that directly connects the molecular network structure (e.g., a specific hydrophobicity) to the resulting PE properties. Unfortunately, standard particle preparation methods often fail in providing suitable particle libraries since varying the network hydrophobicity through using different (co)monomers also affects other colloidal properties like size distribution and network characteristics. Thus, decoupling the influences of network composition and colloidal features is required to establish accurate structure–property relations.

To address this challenge, we have developed amphiphilic nanogels (ANGs) with accurately tuned network hydrophobicity.^50−52^ Since all ANGs originate from one batch of reactive precursor particles, similar colloidal features are obtained and guarantee comparability between ANGs of different hydrophobicity.^52,53^ These nanogels can be functionalized after network formation to form amphiphilic (random) copolymers that contain conventional hydrophilic groups but also pendant hydrophobic units.^53^ Since changing the number of hydrophobic groups allow adjusting the network hydrophobicity precisely, amphiphilic nanogels can be designed to bridge the two worlds of conventional hydrophilic nanogels and solid hydrophobic particles. Using these ANGs as stabilizers for oil/water emulsions enables us to systematically correlate the network hydrophobicity to the properties of the resulting PEs, e.g., the type of emulsion. We find that W/O emulsions are preferentially formed with increasing ANG hydrophobicity, oil polarity, oil/water ratio, and temperature. We were able to connect this behavior to the kinetics of ANG adsorption to the interface and quantify the resulting deformation, i.e., the preferential protrusion of ANGs either in the water or the oil phase. Most importantly, the PEs’ phase inversion behavior can be predicted by the difference in the Flory–Huggins parameter between ANGs with water and with oil (χ_water_ – χ_oil_). Carefully balancing these parameters even allows the formation of W/O emulsions from nonpolar oils, e.g., cyclohexane. Overall, this strategy enabled the determination of crucial structure–property relations that will facilitate the design of new nano/microgels for advanced PEs.

## Results and Discussion

### A Library of Nanogels with Various Hydrophobicity

**Figure 1:**
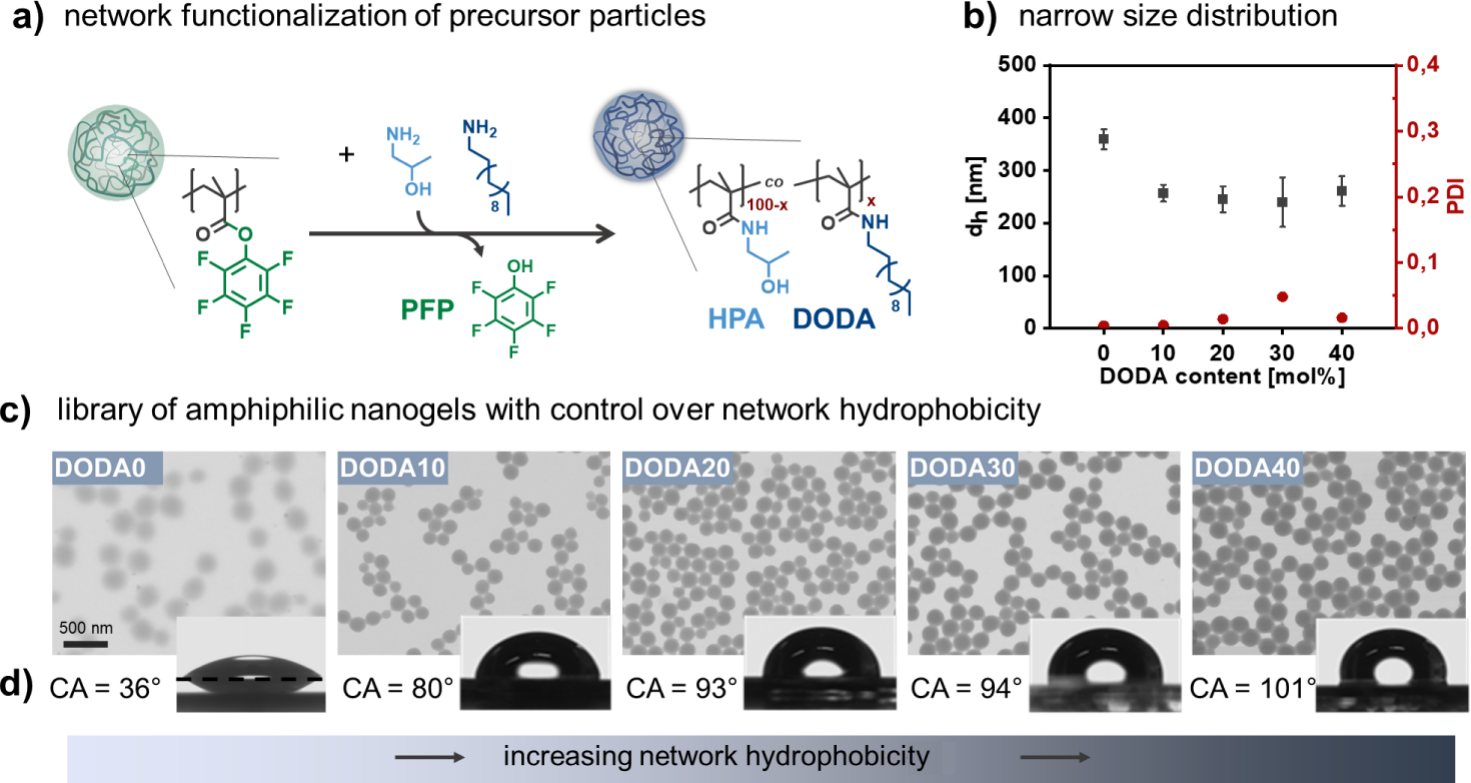
A library of amphiphilic nanogels with varying network hydrophobicity but similar colloidal properties. (a) Synthetic scheme for the preparation of amphiphilic nanogels: Functionalization of reactive networks in PPFPMA precursor particles with mixtures of hydrophilic (HPA) and hydrophobic (DODA) amines. (b) Hydrodynamic diameter obtained by extrapolating angle-dependent DLS data shows similar size for ANGs. Error bars represent the width of the size distribution as full width at half-maximum (fwhm) from measurements at an angle of 90°. Polydispersity indices were also obtained from measurements at an angle of 90° and show narrow size distributions for all ANGs. (c) TEM images of DODA0–DODA40 demonstrate the well-defined colloidal structure of all ANGs. (d) Contact angles of non-cross-linked DODA0–DODA40 polymer films suggest that ANG hydrophobicity increases with DODA content.

Relating nanogel hydrophobicity to PE formation and stabilization requires precise control over the nanogels’ chemical composition, i.e., the ratio of pendant hydrophilic and hydrophobic groups in the network. In standard nanogel preparation methods, this is attempted by varying the ratio between hydrophilic and hydrophobic monomers. However, control is limited due to the different solubility of the monomers. As a result, these processes also change the nanogels’ colloidal structure, preventing the determination of accurate structure–property relations. To overcome these limitations, we have developed a versatile platform for the synthesis of ANGs with varying network hydrophobicity but similar colloidal properties.^50^ This platform uses cross-linked reactive precursor particles that can be transferred into ANGs with defined molecular composition but identical architecture via a postmodification of the network with groups of different hydrophobicity (Figure 1a). Thereby, one batch of precursor particles can give libraries of colloidally similar ANGs with varying chemical network composition.^52,53^

Using this concept, we first prepared well-defined polypentafluorophenyl methacrylate (PPFPMA) precursor particles with a narrow size distribution (*d*_*h*_ = 216 nm, PDI = 0.06) by emulsion polymerization of PFPMA and ethylene glycol dimethacrylate (EGDMA) (1 mol %) as cross-linker. Second, ANGs were synthesized by reacting these precursors with hydrophilic 2-hydroxylpropyl amine (HPA) and hydrophobic dodecylamine (DODA) in varying ratios. To ensure random distribution of both functionalities in the network, functionalization was conducted in DMF as solvent that enables swelling of the polymer network (degree of swelling of 2.8, Figures S2 and S3) and free diffusion of both dissolved reactants HPA and DODA (see Sections 3 and 4 for synthetic details). Since FT-IR suggests quantitative network functionalization (Figure S4) and we assume similar reactivity of HPA and DODA, the feed ratio of HPA to DODA can be used to control the nanogel hydrophobicity. This is supported by control experiments on non-cross-linked polymers that show good agreement between HPA/DODA feed ratio and incorporated ratio (Figure S5). We therefore label the formed ANGs according to the molar ratio of DODA in the network, i.e., DODA0 to DODA40 corresponding to 0 to 40 mol % DODA units in the polymer network (Figure 1a).

Angle-dependent DLS analysis of the nanogels show that size (∼220 nm) and narrow size distribution (PDI < 0.08) of the precursor particles are translated to the ANGs (Figures 1b, S6 and S7). Among the ANGs, DODA0 has the largest diameter. We assign this to the purely HPA-based hydrophilic network, which maximizes swelling in water. TEM images support this observation and show blurry edges for DODA0 as a result of drying the swollen nanogels (Figure 1c). For the other ANGs, TEM also shows a narrow size distribution (Figure 1c) and a homogeneous spherical structure. No microscopic phase separation between hydrophilic and hydrophobic components can be observed.

Having demonstrated control over network composition, we determined how this molecular composition translates to the hydrophobicity of the nanogels. For this, measuring the contact angle of nanogel films is a fast and easy approach. However, on dried colloidal films, surface roughness can give misleading results.^54^ To circumvent such artifacts, we examined the static contact angle on non-cross-linked polymer analogues with similar composition as the ANG networks, i.e., random poly(2-hydroxypropylmethacrylamide-*co*-dodecylmethacrylamide), abbreviated P(HPMA-*co*-DODMA), copolymers containing 0–40 mol % DODA groups (see Section 11 for synthetic details). Contact angle measurements were carried out via the sessile drop technique on homogeneous spin-cast films. The results support our anticipated trend of an increasing hydrophobicity with increasing DODA content. As shown in Figure 1d, the contact angles increase from 36 ± 0° (DODA0) to 101 ± 1° (DODA40). Notably, the increase in contact angle with an increasing fraction of hydrophobic moieties is not linear but exhibits a tipping point, as demonstrated by the drastic increase in contact angle between DODA0 and DODA10 and a less pronounced change in contact angle from DODA20 to DODA30. Thus, hydrophobicity suddenly increased upon reaching a certain fraction of hydrophobic moieties in the amphiphilic copolymers. A similar nonlinear behavior was also observed in our previous work with cholesterol-functionalized nanogels.^55^ These quantitative measures of network hydrophilicity allow us to compare the ANGs to established polymer materials: while DODA0 (pure PHPMA) shows a similar contact angle as established PHPMA films or brushes,^56,57^ the incorporation of 10 mol % DODA groups already increases the hydrophobicity to a contact angle of around 80°. For DODA40, a value of 101 ± 1° suggests a hydrophobic network comparable to polystyrene.^58^ Overall, these results strongly support the amphiphilic character of our nanogels, i.e., they bridge the two areas of soft hydrophilic nanogels and hydrophobic hard particles.

### Amphiphilic Nanogels as Stabilizers for Toluene/Water Pickering Emulsions

**Figure 2:**
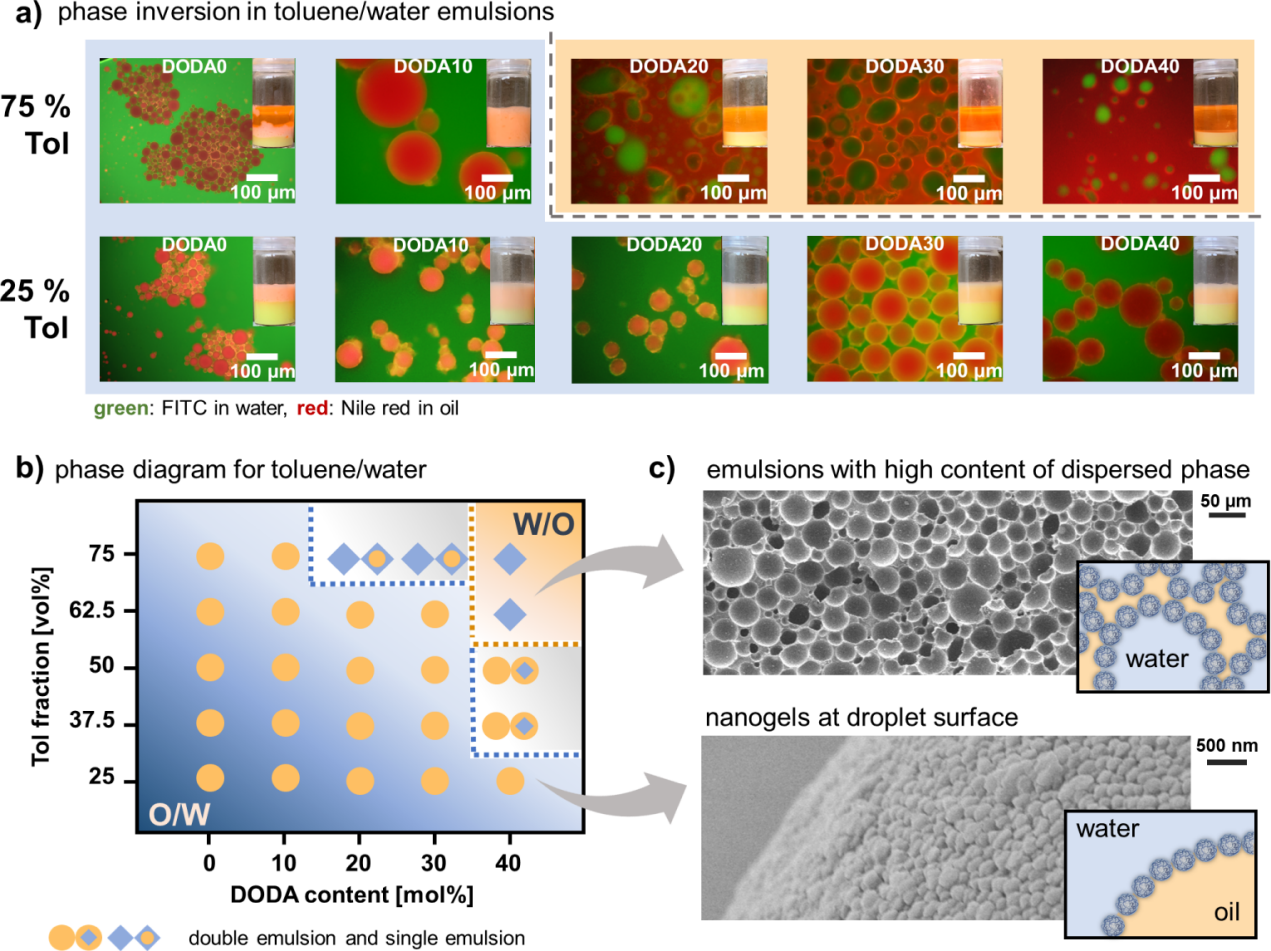
Amphiphilic nanogels can stabilize O/W and W/O emulsions for the toluene/water system. (a) Fluorescence microscopy images and photographs of the emulsions show that for 25 vol % toluene, O/W emulsions are observed for all DODA contents. For 75 vol % toluene an inversion from O/W to W/O is observed between DODA10 and DODA20. (b) Phase diagram of the toluene/water emulsions system summarizing the dependence of emulsion type on the ANGs’ DODA content and the emulsions’ oil fraction. (c) Replacing toluene with styrene enables the solidification of the emulsions by polymerization to visualize the structure of emulsions with high content of dispersed phase (DODA40, Tol-75) and the presence of ANGs (DODA20, Tol-25) at the droplet/particle surface via SEM.

For nanogels as PE stabilizers, emulsion properties are largely determined by the stabilizers’ intrinsic properties, e.g., network hydrophobicity, softness, and size.^22,35,47^ External factors, like oil polarity, oil/water ratio, nanogel concentration, temperature, etc. have a big impact as well.^25,59−61^ Thus, in a first set of experiments we aimed to isolate the influence of the nanogels’ intrinsic network hydrophobicity. For this, we maintained fixed external parameters and used toluene as model oil, a temperature of 20 °C, and a nanogel concentration of 10 mg/mL in the aqueous phase, which we determined to be the lowest concentration to yield stable emulsions (see Figure S8). Thus, the only variable external parameter was the toluene/water ratio. Here, we first examined toluene volume fractions of 25 and 75 vol %, denoted as samples Tol-25 and Tol-75, respectively. For all combinations of nanogel composition and toluene fraction, we examined the resulting emulsion type via fluorescence microscopy using Nile red (NR) and sodium fluorescein (NaFI) to visualize the oil and water phase, respectively. The microscopy images in Figure 2a show a change from oil-in-water (O/W) to water-in-oil (W/O) emulsions by increasing the toluene/water ratio and by increasing the nanogel hydrophobicity. For the low toluene fraction (Tol-25), O/W emulsions are observed for all nanogel stabilizers. This is attributed to the large volume of the water phase (75 vol %) that favors engulfing of the small toluene volume.^33^ In combination with amphiphilic nanogels, this leads to well-defined O/W emulsions that are stable over several weeks (Figure S9). In contrast, a large toluene fraction (Tol-75) should favor the engulfing of water droplets by toluene.^62^ Indeed, at Tol-75, phase inversion to W/O emulsions is observed for nanogels of increased hydrophobicity. Starting with DODA20 and DODA30, mixtures of W/O emulsions and O/W/O double emulsions are formed. For the most hydrophobic DODA40, well-defined W/O emulsions are found.

To further examine the phase inversion phenomenon, we tested additional toluene fractions (Figure S10). From these experiments, a phase diagram was constructed that shows the clear dependence of emulsion type on nanogel hydrophobicity and toluene fraction (Figure 2b). The main trends can be summarized as follows: (1) Hydrophilic DODA0 and DODA10 nanogels are not able to stabilize W/O emulsions even at large toluene fractions, thus indicating a strong interaction between the hydrophilic networks of these ANGs and the water phase. (2) Upon increasing network hydrophobicity (DODA20 and DODA30), W/O emulsions can be formed but require large toluene fractions (Tol-75). (3) For the most hydrophobic nanogels (DODA40), well-defined W/O emulsions can already be formed for smaller toluene fractions (Tol > 50 vol %). At even smaller toluene fractions (32.5 vol % ≤ Tol ≤50 vol %), DODA40 promotes phase transition to less defined W/O/W double emulsions (Figure S10), thus suggesting a strong interaction of these networks with the oil phase. Notably, the DODA40 nanogels do not form aggregates during emulsification (Figure S11). As DODA40 is the most hydrophobic nanogel and therefore most prone to aggregate in the continuous water phase, this result suggests that all nanogels contribute as individual particles to the emulsion stability. This, in turn eliminates potential artifacts of nanogel aggregation on the emulsifying efficiency and allows determining structure–property relations based on the intrinsic nanogel properties. The resulting dense packing of DODA nanogels at the oil/water interface can be visualized by SEM. For these investigations, we exchanged toluene for styrene (25 vol %) which was polymerized after emulsion formation with DODA20 (Figure 2c).

The ANGs’ strong interaction with the oil/water interface enables the stabilization of emulsions with high contents of dispersed phase (>74 vol %). In such emulsions, closely packed droplets cause an increased viscosity.^63^ This is observed for DODA10 at Tol-75. Here, complete emulsification of the toluene/water mixture results in an O/W emulsion with a content of dispersed toluene of 75 vol % (see image of the vial in Figure 2a). By increasing the ANGs’ network hydrophobicity phase inversion occurs. For DODA40, using toluene fractions of Tol-75 and Tol-62.5 gives W/O emulsions with high content of dispersed water phase that coexist with a secondary phase of excess toluene. In the actual emulsion phase, however, the content of the dispersed water phase exceeds 75 vol % (Table S4). This leads to closely packed droplets and an increased viscosity (Figure S12).

Overall, the facile and versatile preparation of such emulsions with high content of dispersed phase (O/W or W/O) is of interest for the development of porous materials^64,65^ for advanced applications such as tissue engineering,^66^ contaminant absorption,^67^ and catalyst support.^68^ To demonstrate this potential, we prepared a solid porous material by simply exchanging the toluene for styrene (75 vol % + AIBN). After emulsification with DODA40 nanogels, we removed the nonincorporated styrene layer from the top of the W/O emulsions with high content of dispersed phase and polymerized the sample by heating. This resulted in a solid porous material (Figure 2c) where the existence of nanogels at the inner pore surface was verified by SEM images (Figure S13). SEM in Figure 2c shows that the size of the packed holes (former water droplets) is similar to the initial water droplets (see Figure S12 for fluorescence microscopy images of the emulsion). Consequently, ANG-stabilized PEs show high stability—even at the elevated temperatures (70 °C) used for polymerization.

### Interfacial Tension and Nanogel Deformation Depend on Nanogel Hydrophobicity

**Figure 3:**
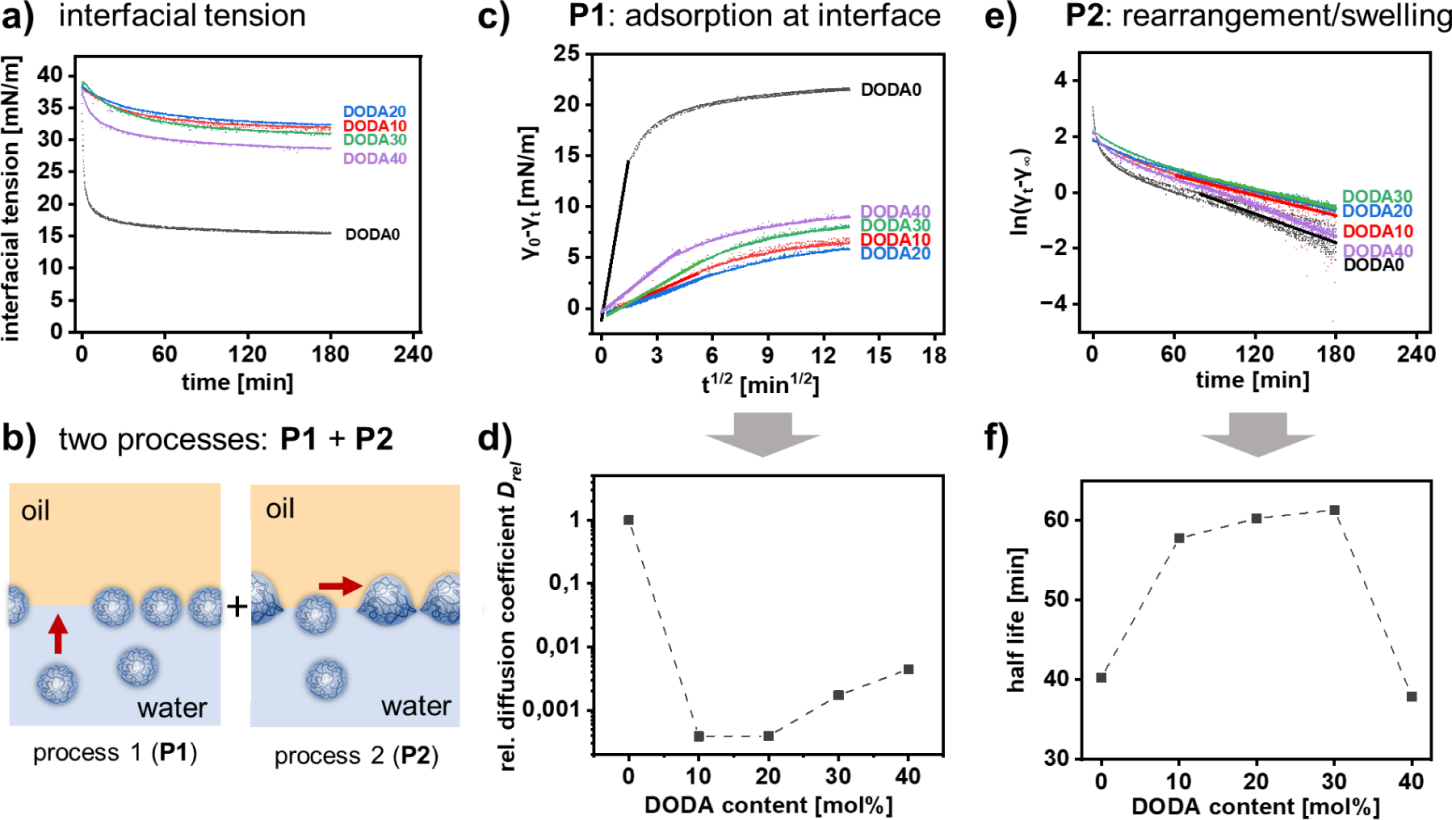
Interfacial tension (IFT) between toluene and water depends on ANG hydrophobicity and time. (a) IFT decreases nonlinearly over time with varying rates among different ANGs. (b) Two different processes influence the IFT kinetics: adsorption of ANGs to the interface (P1) and rearrangement and swelling of ANGs at the interface (P2). (c) Analysis of P1 from the early stages of IFT reduction gives access to a diffusion coefficient that describes the rate of ANG adsorption to the interface. (d) The relative diffusion coefficient that describes interfacial adsorption (w.r.t. DODA0) depends on the ANGs’ DODA content: Hydrophilic DODA0 and hydrophobic DODA40 show fastest adsorption to the interface. (e) Analysis of P2 from the late stages of IFT reduction gives access to a half-life that describes the rate of ANG rearrangement. (f) Half life depends on DODA content: Hydrophilic DODA0 and hydrophobic DODA40 show fastest rearrangement.

Different toluene/water emulsions (O/W or W/O) result from the adsorption of ANGs with varying network hydrophobicity to the O/W interface. To examine this in more detail, we determined the interfacial tension (IFT) of the toluene/water system for the different ANGs. Note that the hydrophobic nanogels do not undergo interphase transfer from the water phase to the oil phase (Figure S14). Therefore, this analysis only considers the adsorption from the water phase to the oil/water interface, neglecting any potential adsorption of nanogels from the oil phase to the interface. For all samples, DODA0–DODA40, IFT decreases with time (Figure 3a). This contrasts with solid particles, which do not show a strong reduction in IFT,^31,69^ and indicates the distinctive combination of colloidal and polymeric features of the soft ANGs. Upon interaction with the interface, these flexible amphiphilic colloids deform to maximize their contact with oil and water, thus decreasing the interfacial tension.^70−72^

Closer inspection of the IFT kinetics shows two distinct processes for all nanogels: an initial rapid decrease (P1), and a subsequent slow relaxation to the final equilibrium state (P2) (Figure 3b). We assume that P1 can be attributed to the fast adsorption of ANGs from the aqueous dispersion to the interface.^71,73,74^ For P2, we suggest that the dominating effects are rearrangement of nanogels at the interface due to spreading and conformational change of polymer chains, or formation of a nanogel sublayer near the interface.^73−76^

To quantify the contribution of the two processes, specific physical parameters can be extracted from the kinetic data. For P1, a diffusion coefficient *D* can be determined to describe the rate of ANG adsorption to the interface. This can be obtained from linearly fitting the initial stages in a graph of (γ_0_ – *γ*_*t*_) vs *t*^1/2^ (Figure 3c and see Section 23 and Table S5 for calculation details).^73,74^ Correlating the relative rates *D*_*rel*_ (w.r.t. to hydrophilic DODA0) against network hydrophobicity shows that the ANGs adsorb with different velocity to the interface (Figure 3d). Interestingly, this change in adsorption rate does not follow a monotonic increase or decrease from DODA0 to DODA40. Instead, *D*_*rel*_ first decreases from DODA0 to DODA10–20 and then increases again for DODA30–40. In general, a high rate of adsorption suggests a high affinity of nanogels to the interface, which is assumed to correlate with ANG deformation.^71^ Thus, we suggest that the soft, hydrophilic DODA0 nanogels quickly adsorb to the interface where they strongly deform due to their preferential interaction with the water phase. In contrast, we assume that the more hydrophobic DODA40 nanogels adsorb rapidly due to their preferential interaction with the toluene phase, which causes strong deformation by swelling in the oil phase. In comparison to the hydrophilic and hydrophobic ANGs, the amphiphilic nanogels (DODA10–DODA30) show no clear preference for either phase, thus exhibiting a lower rate of adsorption and reduced deformation at the interface.

For the second process P2, a half-life can be defined as the rate that is required to fully rearrange the network at the interface and reach IFT equilibrium.^73,75^ The respective values can be determined from linearly fitting the last section of a plot of ln(*γ*_*t*_*– γ*_*∞*_) against *t* (Figure 3e and see Section 23 for calculation details).^73^ The dependency of half-life on the nanogel hydrophilicity shows a similar trend as the diffusion rate of P1. DODA0 and DODA40 show the shortest half-lives of around 40 min. In contrast, DODA10–DODA30 exhibit longer half-lives of around 60 min, i.e., the networks of DODA10–DODA30 rearrange slower than the polymer segments in the DODA0 and DODA40 nanogels. This supports the suggested differences in interaction between nanogels and oil/water phase and is in line with the respective nanogel deformation of P1.

For different nanogel hydrophobicities, we hypothesize that the different kinetics of interfacial adsorption and rearrangement will also affect the nanogels’ ability to stabilize emulsions. We therefore measured the adsorption efficiency of the different nanogels by determining the fraction of interfacially adsorbed nanogels after emulsification, using toluene/water emulsions (50/50 vol/vol) as model systems. We extracted a fixed amount of the continuous water phase from the bottom (toluene emulsion droplets cream to the top) and determined the mass of remaining, nonadsorbed nanogels gravimetrically after freeze-drying (see Section 23 for detailed methods and discussion). This allows determining the fraction of interfacially adsorbed nanogels from the total amount of available nanogels (note that nanogels do not transfer into the toluene phase, see Figure S14). The results, shown in Figure S15, suggest that the nanogels with intermediate hydrophobicity (DODA20 and DODA30) adsorb less to the interfaces (around 25%) than the more hydrophilic and hydrophobic counterparts DODA0 and DODA40 (around 50%). We corroborate this experimental assessment by a simple mathematical estimation of the adsorption efficiency from the average droplet size and the size that a single nanogel occupies at the interface. The results qualitatively reproduce this trend (see Figure S16 and Section 23 for detailed methods and discussion). Our finding that nanogels with intermediate hydrophobicity adsorb less than their more hydrophilic and hydrophobic counterparts is consistent with their slower interfacial adsorption and rearrangement kinetics (Figure 3).

**Figure 4:**
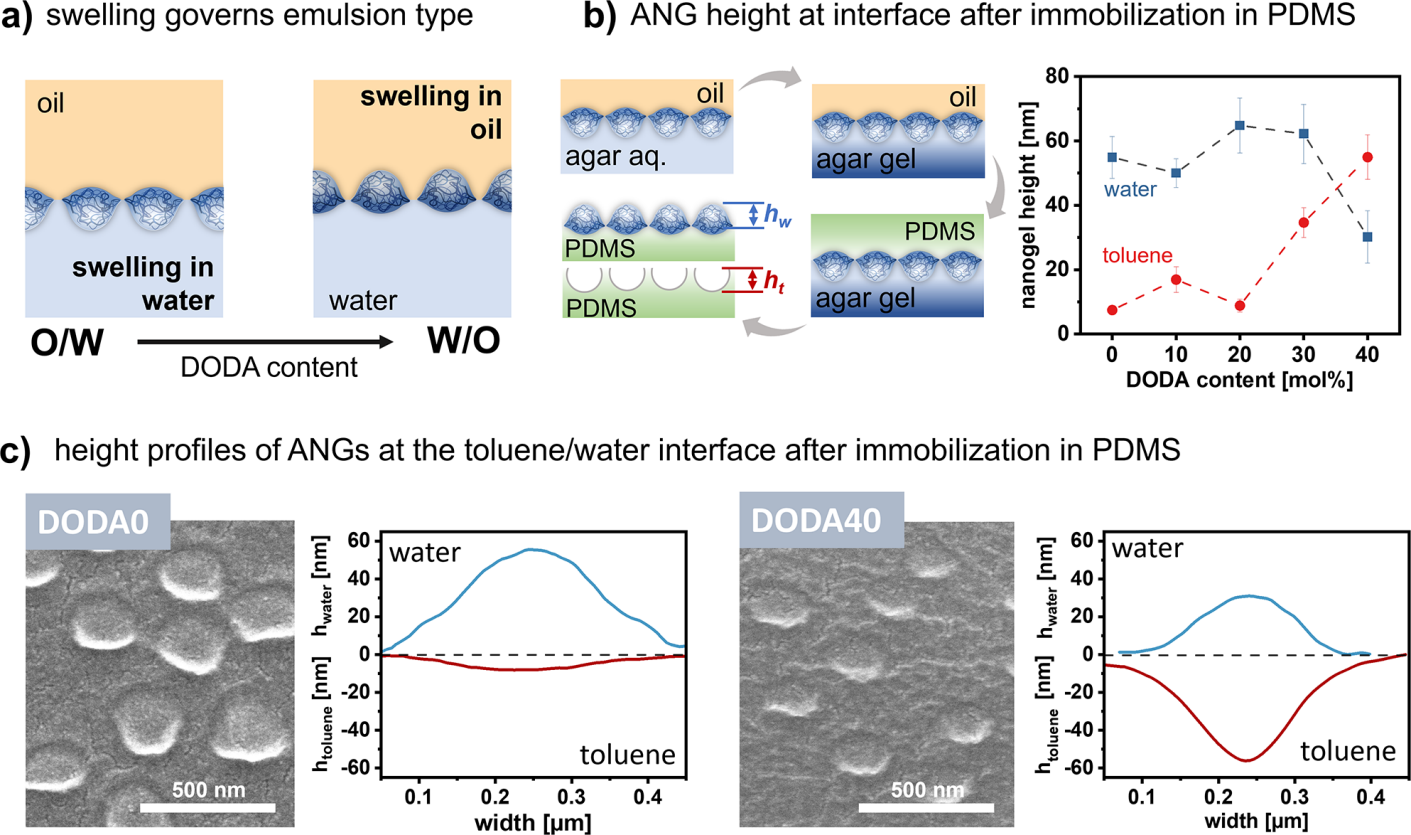
Swelling, location, and deformation of ANGs at the toluene/water interface. (a) Schematic representation to illustrate how ANG swelling/deformation at the oil/water interface governs the emulsion type. (b) ANG protrusion heights in oil and water indicate that DODA0–DODA30 protrude more into the water phase, while DODA40 protrudes more into the toluene phase. The height in water was determined by measuring immobilized ANG particles at the surface of a PDMS gel matrix. The height in toluene was determined from the cavities after removal of ANG particles from the PDMS. (c) Representative SEM images of nanogels on the surface of the PDMS gel matrix show DODA0 protrudes more in the water phase while DODA40 more in the oil phase. Representative height profiles of nanogels from AFM images demonstrate both DODA0 and DODA40 deform at toluene/water interface: DODA0 exhibits increased swelling in the water phase, whereas DODA40 shows increased swelling in the toluene phase.

ANG deformation could explain the observed shift from O/W emulsions for DODA0 to W/O emulsions for DODA40 (at Tol-75). Since the network hydrophobicity is coupled to a solvent preference, we suggest that the ANGs follow Finkle’s rule,^42^ i.e., hydrophilic DODA0 protrude to the water phase, while more hydrophobic DODA40 protrude to the toluene phase (Figure 4a). To test this hypothesis, we examined the nanogels’ position at the toluene/water interface via the gel trapping technique^77^ (see Section 24 for details). For this, we replaced the water phase by a 2 wt % agarose solution, which remains fluid at elevated temperature but gels when cooled to room temperature. To provide sufficient time for the nanogels to relax into their equilibrium position, we ensure a long equilibration time (3 h) and a slow cooling (1–2 h) to room temperature. With this process, we hypothesize that the final nanogel position resembles that of nanogels directly equilibrated at room temperature. Exchanging the oil phase to a polydimethylsiloxane (PDMS) polymer precursor solution allows transferring the interfacially adsorbed ANGs to a solid surface after hardening the PDMS (Figure 4b). Examination of these surfaces via AFM gives access to the height profile of the ANGs in the water phase. In addition, removing the ANGs from the PDMS with ethanol or adhesive tape produces cavities whose depth represents the ANG protrusion into the oil phase (Figure 4b; see Section 25 for measurement details). As can be seen from Figure 4b, the ANG height in the water phase decreases from DODA0–DODA40 while the height in the toluene phase increases. AFM height profiles and SEM images representing the ANG protrusion into the water phase (Figure 4c) support this trend: hydrophilic DODA0 protrudes more into the water phase (∼55 nm) than the oil phase (∼8 nm) whereas hydrophobic DODA40 protrudes more into the oil phase (∼55 nm) than the water phase (∼30 nm). We assume that this strong deformation is part of the driving force for their fast interfacial adsorption (P1) and rearrangement (P2). In contrast, ANGs of medium hydrophobicity (DODA10–DODA30) reside at intermediate positions of the oil/water interface (Figures 4b, S17–S21), thus showing similar affinity to water and toluene. We suggest that the limited deformation is causing a comparably slow interfacial adsorption (P1) and rearrangement (P2).

Overall, these findings demonstrate the distinctive structure of ANGs that combine properties of small surfactants, solid particles, and soft nano/microgels. First, parallels between network hydrophilicity of ANGs and the HLB (hydrophilic–lipophilic balance) of traditional surfactants can be drawn: For hydrophilic small molecules, a high HLB also leads to O/W emulsions while hydrophobic surfactants with a low HLB also generate W/O emulsions.^32,78,79^ Second, similarities between ANGs and solid particles become obvious: In traditional PEs, emulsion type also depends on particle wettability, i.e., O/W for hydrophilic and W/O for hydrophobic particles.^1^ In addition, the actual process of emulsification can be inefficient for particles of intermediate amphiphilicity (i.e., a contact angle of ca. 90°) while hydrophilic particles and hydrophobic particles perform better.^33,80^ Similarly, ANGs with medium hydrophobicity were found to exhibit slower interfacial adsorption than their hydrophilic and hydrophobic counterparts, thus demonstrating the influence of kinetics on emulsion formation. Third, ANGs behave like traditional micro/nanogels, where the adsorption kinetics of ANGs to the oil/water interfaces, the ANG interfacial deformation and the affinity of ANGs to water and oil all influence the resulting interfacial tension.^71,75,81^

### Stabilization of Various Oils and Prediction of PE Type by Flory–Huggins Parameters

**Figure 5:**
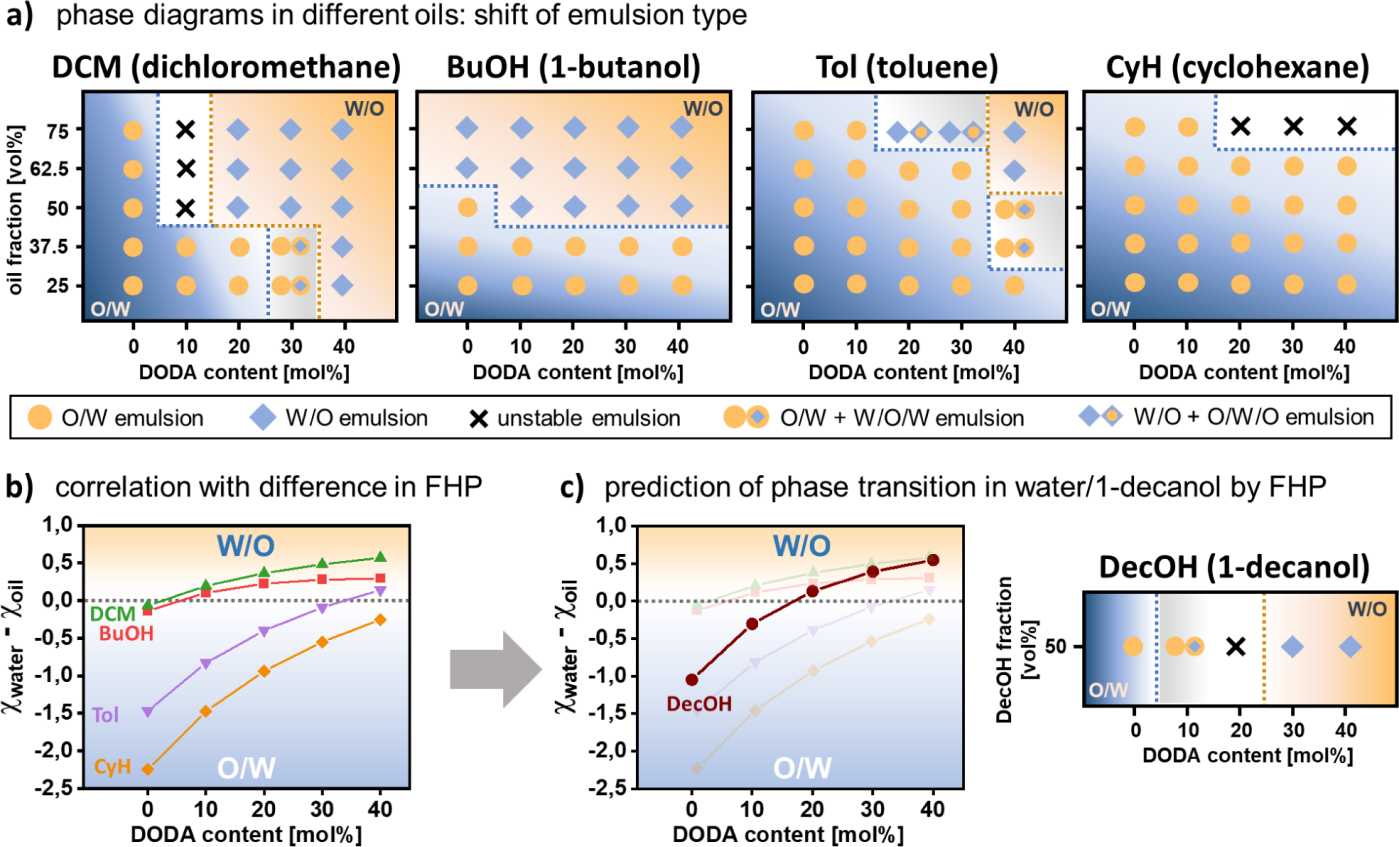
Flory–Huggins parameters allow prediction of phase inversion in different oil/water emulsion systems. (a) Phase diagrams of dichloromethane/water, 1-butanol/water, toluene/water, and cyclohexane/water emulsions show a shift in phase inversion depending on the oil. Here, orange circles indicate O/W emulsions, blue diamonds indicate W/O emulsions, crosses indicate unstable emulsions, orange circles plus blue diamonds within orange circles indicate O/W emulsions including W/O/W droplets, and blue diamonds plus orange circles within blue diamonds indicate W/O emulsions including O/W/O droplets. (b) The difference of calculated Flory–Huggins parameters between ANG and water and ANG and oil (*χ*_*water*_ – *χ*_*oil*_) indicates preferable interaction of ANGs with either water or oil phase (c) For 1-decanol, calculation of *χ*_*water*_ – *χ*_*oil*_ = 0 can predict the phase inversion point, i.e., the DODA content at which O/W emulsions shift to W/O emulsions.

To demonstrate the versatility of ANGs as stabilizers, we prepared emulsions with 4 different oils of decreasing polarity, i.e., 1-butanol (BuOH), dichloromethane (DCM), toluene (Tol), and cyclohexane (CyH). For each system, we determined the emulsion type as a function of ANG hydrophobicity (i.e., DODA content) and volume fraction of oil. The resulting phase diagrams (Figures 5a, Figure S10 and S22–S24 for fluorescence microscopy images) show pronounced changes between the different oils. Most importantly, the region of phase inversion between O/W and W/O is shifting. With increasing polarity of the oil, W/O emulsions are formed more readily, i.e., with more hydrophilic ANGs and for lower volume fractions of oil. For example, in the case of DCM and BuOH, W/O emulsions are already observed for relatively hydrophilic ANGs with DODA contents of 10–20 mol % at low oil fractions of 50 vol %. In contrast, the less polar toluene requires higher DODA contents of 30–40 mol % and a high toluene fraction of at least 62.5 vol %. For the nonpolar cyclohexane, no stable W/O emulsions are observed at room temperature even for the most hydrophobic ANGs and the highest oil fractions.

To further elucidate the emulsification ability of the different nanogels, we measured the droplet sizes of the different oil/water emulsions, all with a 50/50 volume ratio (Figure S26 and see Section 26 for detailed discussions). We found that the emulsion droplet size decreases with increasing oil polarity, which correlates with the lower IFT of the oil/water systems^82^ (details in Supporting Information Section 26 and Figure S26). The influence of nanogel hydrophobicity on the droplet size for polar oils is not pronounced, while for nonpolar oils, DODA0 nanogels produce the smallest emulsion droplets and thus seemingly are the most efficient stabilizers, in agreement with the fastest adsorption kinetics and the lowest IFT of toluene/water with DODA0 (Figure 3), as discussed above. We also noticed that the emulsion type does not have an obvious influence on the droplet size, which remains similar for DCM/water and 1-butanol/water emulsions and their corresponding W/O analogues (Figure S26).

It is obvious that ANGs can stabilize both O/W and W/O emulsions for different oils. Based on the changes in interfacial shape of the ANGs (Figure 4), we assume that phase inversion is connected to the DODA-dependent solvent preference of the ANGs: If swelling in water is more prominent, O/W emulsions are favored. Conversely, if swelling in the oil phase is preferred, W/O emulsions are formed. This hypothesis is supported by the ANGs’ degree of swelling (DGS) in different oils (Figure S27). Here, swelling in water decreases with increasing DODA content, while swelling in oil (BuOH or toluene) increases. Similarly, water solubility of non-cross-linked P(HPMA-*co*-DODMA) copolymers decreases with increasing DODA content while solubility in BuOH or toluene increases (Figure S28).

While these results indicate a clear influence of network composition on the ANGs’ interaction with water and oil, they remain a qualitative assessment. This can be attributed to experimental limitations. Coagulation of ANGs in nonpolar oils can distort the DGS and polymer swelling can hinder accurate determination of solubility. Thus, it remained difficult to predict the PE type for a given DODA content and oil. We address this limitation via a quantitative measure for the interaction between ANGs and oil and water. A suitable measure is the use of Flory–Huggins parameters (*χ)*. These numerical values can describe the interaction of (co)polymers with different solvents and are easily calculated from the Hansen solubility parameters of polymer and solvent.^83−86^ In contrast to established approaches that use oil polarity^36,80,87^ or solubility parameters^47^ as numerical values, χ also includes the influence of dispersion forces and hydrogen bonds. Especially for amphiphilic nanogels, these contributions must not be neglected. To provide a quantitative prediction of PE type, we calculated χ for the interaction between the different ANG networks and water and oil, giving *χ*_*water*_ and *χ*_*oil*_ for each DODA content (see Section 29 for calculation details). Then, the preference of a specific ANG for either water or oil phase could be described as the difference between both values, *χ*_*water*_ – *χ*_*oil*_. Since a low χ value indicates a good interaction,^83,84^ a negative value for *χ*_*water*_ – *χ*_*oil*_ suggests a preferred interaction with the water phase (since *χ*_*water*_ < *χ*_*oil*_). In contrast, a positive value (*χ*_*water*_ > *χ*_*oil*_) indicates preferred interaction with the oil phase. Consequently, we suggest that a switch from negative to positive values indicates a shift in the preferred emulsion type. Thus, *χ*_*water*_ – *χ*_*oil*_ = 0 should indicate the threshold for phase inversion from O/W to W/O—at least if the volume fraction of oil is not considered, i.e., at 50/50 oil/water ratios.

To test this hypothesis, we plotted *χ*_*water*_ – *χ*_*oil*_ against the DODA content of the ANGs (Figure 5b). For all oil/water pairs, *χ*_*water*_ – *χ*_*oil*_ increases with network hydrophobicity, thus indicating an increasing interaction of the ANGs with the oil. For different oils, *χ*_*water*_ – *χ*_*oil*_ becomes zero at different DODA contents. At oil fractions of 50 vol %, these values correlate very well with the experimentally determined phase inversion points (Figure 5a). For the DCM/water and the BuOH/water systems, the calculated values suggest phase inversion between DODA0 and DODA10. This is in line with the experimentally observed phase inversions between DODA0 and DODA20 for DCM and between DODA0 and DOA10 for BuOH. For toluene, the calculations suggest phase inversion between DODA30 and DODA40, which is consistent with experimental observations. Finally, for cyclohexane, *χ*_*water*_ – *χ*_*oil*_ is negative for all DODA contents, in agreement with the occurrence of O/W emulsion for all ANGs.

These findings suggest the potential to predict phase inversion for other oils. To test the predictive power of our approach, we calculated *χ*_*water*_ and *χ*_*oil*_ for 1-decanol. The resulting graph in Figure 5c shows that *χ*_*water*_ – *χ*_*oil*_ becomes zero between DODA10 and DODA20, thus suggesting phase inversion. Indeed, the experimental system shows a clear shift from O/W for DODA0 to W/O for DODA30 (Figure 5c and see Figure S25 for fluorescence microscopy images). Between these defined phases, poor emulsion stability and W/O/W double emulsions occur. This suggests a limited preference of the ANGs for either phase at this composition.

### Influence of Emulsification Temperature on Phase Inversion and Metastable Emulsions

**Figure 6:**
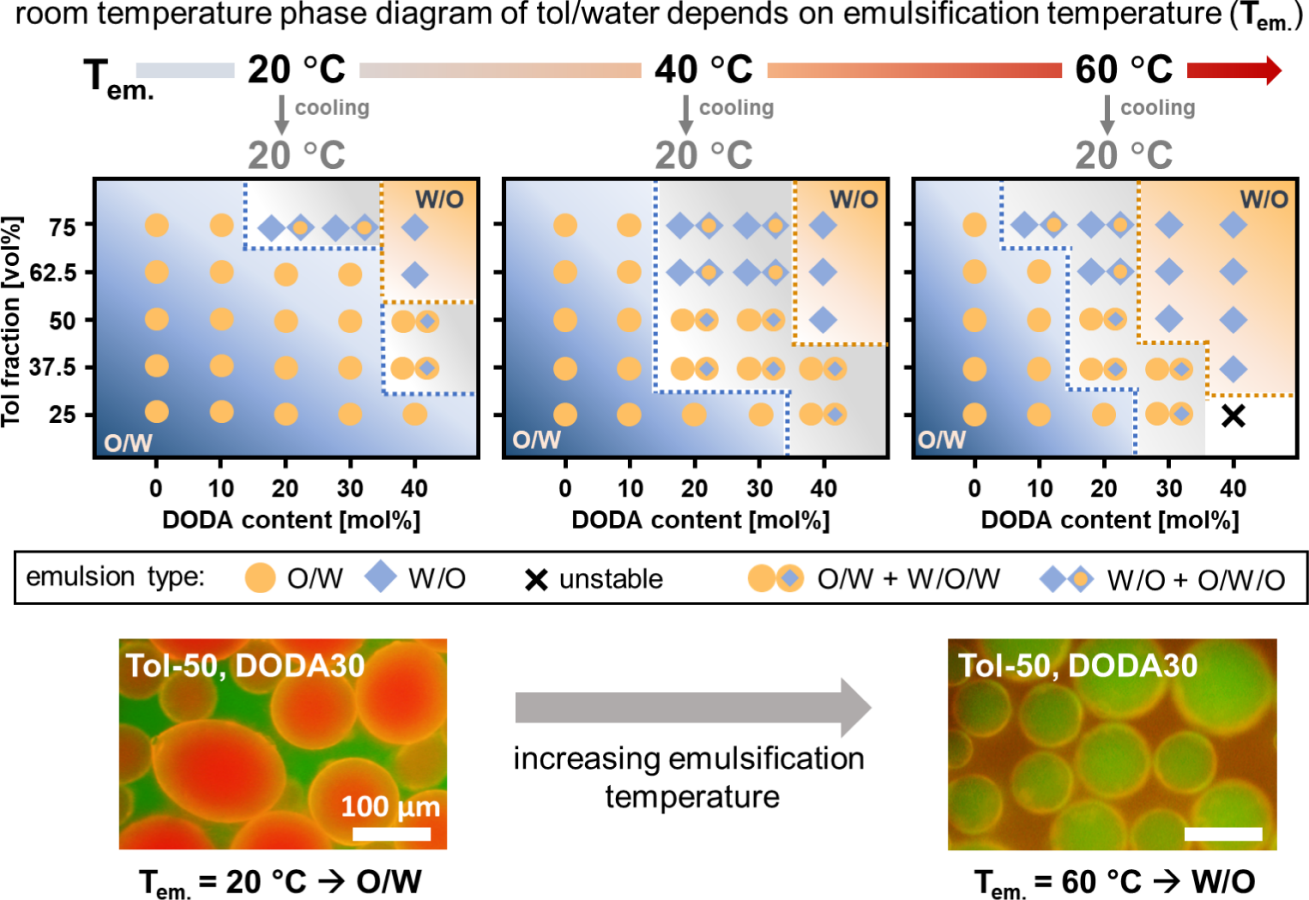
For toluene/water (tol/water) emulsions, increasing emulsification temperature (*T*_*em.*_) gives access to phase inverted metastable emulsions at room temperature. At higher emulsification temperatures, the border between O/W and W/O emulsions shifts and W/O emulsions are observed at lower DODA contents and lower oil fractions. Here, orange circles indicate O/W emulsions, blue diamonds indicate W/O emulsions, crosses indicate unstable emulsion, orange circles plus blue diamonds within orange circles indicate O/W emulsions including W/O/W droplets, and blue diamonds plus orange circles within blue diamonds indicate W/O emulsions including O/W/O droplets.

Competitive interactions between the ANGs and the oil and water phase determine the emulsion properties. Since these interactions are temperature-dependent, phase inversion should also depend on temperature. To test this assumption, we prepared toluene/water emulsions at emulsification temperatures (*T*_*em.*_) of 20 °C, 40 °C, and 60 °C and investigated the resulting emulsion type after cooling the emulsions to room temperature (see Figures S10 and S29 for fluorescence microscopy images). As shown in Figure 6, a clear influence of emulsification temperature on the (room temperature) phase diagram can be observed. With increasing *T*_*em*__._, the border between the phases shifts toward lower DODA contents and lower toluene/water ratios. Thus, W/O emulsions are more prevalent when emulsions are prepared at high temperatures. For example, at *T*_*em.*_ = 60 °C, W/O emulsions can already be formed with DODA30 at 50 vol % toluene (see Figure 6). This could be assigned to either a reduced interaction between ANGs and water or an increased interaction between ANGs and toluene. Since temperature-dependent DLS measurements do not show any influence of temperature on ANG swelling in water (Figure S30), we suggest that an increased affinity for the toluene phase is the driving force in this system, as indicated from the increased swelling of nanogels in toluene at higher temperature (Figure S31). Importantly, this increased affinity is only required during the emulsification step. After the successful formation of W/O emulsions at elevated temperatures, all these systems remain as stable emulsions at room temperature. This surprising stability suggests that the ANGs’ strong interfacial adsorption keeps them kinetically trapped at their position even after cooling to room temperature. As a result, this process allows preparing metastable W/O emulsions that are not accessible under our standard emulsification conditions at room temperature.

**Figure 7:**
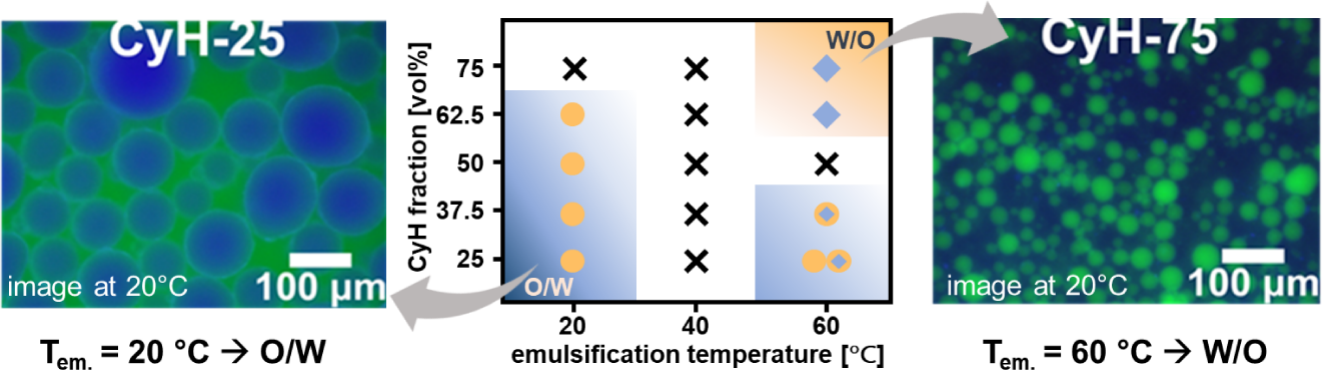
Temperature-mediated control over emulsion type. Using DODA40 at higher emulsification temperatures enables the formation of W/O emulsions from nonpolar cyclohexane. Exemplary fluorescence microscopy images and an emulsification temperature dependent phase diagram show the shift from O/W to W/O emulsion between 20 and 60 °C. In this study, CyH was labeled with anthracene, exhibiting a blue color, while water was labeled with fluorescein sodium, displaying a green color.

We capitalize on the ability to control the solvent-ANG interactions via temperature to form metastable emulsions that are otherwise instable at room temperature. We use the example of cyclohexane as a highly nonpolar oil that does not form W/O emulsions at room temperature, even with the most hydrophobic DODA40 (Figure 5). By increasing the emulsification temperature to 60 °C, W/O emulsions are formed, presumably due to the increased ANG affinity to the oil phase at higher temperature, while W/O emulsions persist at room temperature (Figure 7, see Figure S32 for fluorescence microscopy images). Thus, a metastable emulsion type that is not accessible with room temperature-emulsification can be prepared by exploiting the temperature-depending ANG interfacial properties.

The versatility of this approach becomes obvious when considering other nonpolar oils that are difficult to formulate into W/O emulsions, e.g., rapeseed oil. At *T*_*em.*_ = 60 °C, DODA contents as low as DODA20 already enable the preparation of well-defined W/O emulsions at low rapeseed oil fractions of 50 vol % (see Figure S33 for fluorescence microscopy images). Since all these emulsions remain stable after cooling to room temperature, this strategy drastically increases the potential of ANGs as versatile stabilizers for PEs.

## Conclusions

We successfully established a quantitative correlation between nanogel structure and the type of stabilized Pickering emulsion. Employing a postfunctionalization protocol on precursor nanoparticles, we were able to create a library of nanogels with precisely tunable structure and hydrophobicity, while maintaining uniform colloidal features such as size and size distribution.

We developed a quantitative model to predict emulsion types for a broad spectrum of oils. This model uses Flory–Huggins parameters (FHP) to quantify the underlying interaction dynamics between the nanogel network and both water and oil phases. In comparison to other methods, the FHP approach stands out by considering the full spectrum of interactions—dispersion forces, polar interactions, and hydrogen bonding. Previous studies that correlate emulsion type solely with oil polarity can overlook the critical role of the dispersion component, particularly in nonpolar solvents. Also, these approaches can underestimate the significance of hydrogen bonding, especially in the presence of strong agents like alcohols. At the same time, studies that attribute emulsion type to the solubility parameters of polymers neglect the influence of molecular volume on the polymer–solvent interaction. Since this affects the molar concentration of the solvent, the mixing entropy between the polymer and solvents is overlooked. Therefore, we see the FHP method as a comprehensive approach that addresses these gaps, thus offering a more nuanced understanding of the factors governing PE behavior. Further studies in this area could greatly benefit from systematic FHP calculations of polymers in various solvents.

While ANGs share characteristics with solid nanoparticles, they also resemble the behavior of molecular surfactants as they can decrease the oil/water interfacial tension. To understand the influence of the ANGs’ structure on the emulsion properties, we examined the interfacial adsorption kinetics in detail. For ANGs of increasing network hydrophobicity, we discovered that the equilibrium interfacial tension and its decrease rate correlate with the nanogel’s swelling behavior, deformation capacity, and positioning at the oil/water interface. Here, nanogels with intermediate amphiphilicity show the slowest interfacial absorption and deformation, thus indicating a poor emulsification capacity. However, once formed, these PEs are assumed to be highly stable due to the significant energy required to detach ANGs from the interface. This discrepancy highlights the need to differentiate between emulsification ability and postformation stability of emulsions, concepts that are often conflated in the literature. Finally, as for molecular surfactants, emulsification with ANGs is temperature-dependent. For highly nonpolar solvents, emulsification at elevated temperatures allows the formation of W/O emulsions that remain stable when cooling to room temperature. Our study suggests that the affinity of the ANGs to the oil phase increases with temperature, leading to a phase inversion upon emulsification. The strong interfacial adsorption of the ANGs subsequently kinetically traps the nanogels at their location after cooling the system, thus resulting in metastable emulsions that persist at room temperature, which are otherwise not accessible. It is our hope that this demonstration of temperature as a transient parameter to influence the emulsion behavior triggers more extensive computational and experimental research for the preparation of otherwise inaccesible emulsions. Interesting challenges include the quantification of the ANGs’ temperature-dependent affinity to oil and water, the resultant interfacial position and reduction of interfacial tension, and, finally, the dynamics of such interfacial microgels upon heating and cooling.

Overall, the developed structure–property relations show the versatility of amphiphilic nanogels as stabilizers for Pickering emulsions. This holds promise for their tailor-made application in diverse fields, i.e., cosmetics, pharmacy, interfacial catalysis, and beyond.

## Methods

### Nanogel Preparation and Characterization

The nanogels with different hydrophobic dodecyl (DODA) contents were prepared by postfunctionalizing the precursor poly(pentafluorophenyl methacrylate) (PPFPMA) nanoparticles, via the active ester substitution reaction according to literature.^50^ The detailed synthetic methods can be found in the Supporting Information Section 24. Briefly, in a typical reaction, 2 g (7.93 mmol PFPMA units) of the freeze-dried PPFPMA precursor particles were dispersed in 150 mL DMF under ultrasonication. A mixture of dodecyl amine and 2-hydroxylpropyl amine (see Table S1 for the corresponding amounts) was subsequently added. Then 3.310 mL triethylamine (TEA) (23.64 mmol) as base was added. The mixture was allowed to react for 6 days at 60 °C. The resulting nanogels were purified by extensive dialysis against DMF and then against water. Note that the volume of the dispersion inside the dialysis tube expands 2-fold when changing from DMF to water. The purified nanogel dispersion was concentrated by centrifugation and redispersion in water. Finally, the concentration of the nanogel dispersion was gravimetrically determined after lyophilizing 1 mL of the dispersion. The hydrodynamic diameter of nanogels in water was measured by dynamic light scattering (DLS) with a Nicomp Nano Z3000 system. The samples were sonicated before measurements. The transmission electron microscopy (TEM) images of nanogels were obtained by the Hitachi FE-SEM SU8030 with the acceleration voltage of 30 kV and current of 10 μA.

### Emulsification and Fluorescence Microscopic Studies

To form Pickering emulsions, varying volumes of nanogel dispersion and organic solvent were combined to give a total volume of 4 mL in a glass vial (5 mL capacity). The concentration of nanogels in water was 10 mg/mL (1 wt %) and the dispersion was dyed with fluorescein sodium salt (0.1 mg/mL). The oil phases contained 0.03 mg/mL of Nile red (for toluene, DCM, 1-butanol, and 1-decanol) or 0.5 mg/mL of anthracene (for cyclohexane) due to poor solubility of Nile red in cyclohexane. The mixture was then subjected to cycles of ultrasonication in an ultrasonic bath for 1 min followed by vortex mixing for 10 s, repeated 10 times. The ultrasonic bath was also capable of heating to 40 and 60 °C to create emulsions at elevated temperatures. For the fluorescence microscopy investigations, the Pickering emulsion was first diluted in the corresponding continuous phase and then placed on a glass slide with a concave dent in the middle, covered with a cover slide and measured under a Keyence BZ-X810 fluorescence microscope.

### Interfacial Tension Between Toluene and Water with Nanogels

Before the measurements, all nanogel samples were extensively purified. Freeze-dried DODA10 to DODA30 samples were redispersed in DMF and washed with DMF via centrifugation (10k rpm, 2 h) and redispersion for 3 times. Afterward, the samples were extensively dialyzed against water. For DODA40, 1-butanol was used for redispersion and washing in a similar procedure. Then, the DODA40 dispersion was first dialyzed against DMF to remove 1-butanol and then against water. All samples were concentrated by centrifugation and redispersion in water. The interfacial tension (IFT) was measured by the pendant drop method. For this, a 50 μL drop of the aqueous nanogel dispersion (10 mg/mL) was suspended in the toluene (HPLC grade) in a quartz cuvette. IFT data were recorded at a constant rate of 10 points/min to study the kinetics of IFT decrease.

### Gel-Trapping Experiments to Fix Nanogels at Oil/Water Interfaces

To observe the nanogel positioning at the oil/water interface, we employed the gel-trapping technique to immobilize the nanogels.^77^ The detailed methods can be found in the Supporting Information. Briefly, first, 2 mL 20 mg/mL agarose aqueous solution was prepared at 90 °C in an oil bath. Second, 1 mL of toluene, preheated to 90 °C, was gently layered atop the agarose solution. Third, the vial was transferred to a 60 °C water bath to maintain the agarose in solution form and left undisturbed for 30 min to achieve thermal equilibrium. Fourth, 100 μL nanogel dispersion in ethanol/water mixture (1/1 vol/vol) with a concentration of 500 μg/mL was gently introduced to the toluene-water interface using a micropipette. The system was allowed to equilibrate for 3 h to facilitate the equilibration of nanogels at the interface. Finally, the vial was set aside to cool to room temperature to trigger the gelation of the agarose.

After gel formation, the toluene supernatant was carefully decanted from the vial. The remaining gel was covered with a roughly 5 mm thick layer of Polydimethylsiloxane (PDMS) containing curing agent in a mass ratio of 10:1. The system was left to solidify for 48 h. Subsequently, the PDMS layer was peeled from the gel phase and the residual agarose was removed by immersing the PDMS disc in water (90 °C) for 10 min and rinsing with water, repeated three times. The PDMS film was then air-dried and coated with a thin gold film (∼10 nm) if needed to improve scanning electron microscopy (SEM) imaging. AFM measurements were used to capture the original protrusion of nanogels in both water and oil phase, by scanning the nanogels on the PDMS gel surface and the cavities formed after nanogel removal—accomplished by mechanical rubbing for DODA0 and by an adhesive tape for DODA10–DODA40.

## Data Availability

The data that support the findings of this study are available via Zenodo, DOI: 10.5281/zenodo.13235695 or from the corresponding author upon request.

## Supporting Information Available

Information on materials and instrumentation, synthetic procedures and characterization data for monomer, nanogels, and control polymers (NMR, FT-IR, GPC, DLS, TEM, contact angle, nanogel size in different solvents), procedure for preparation of Pickering emulsions and their characterization (fluorescence microscopy, droplet size, stability), fluorescence microscopy images of phase diagrams for different oils (dependence on nanogel composition and oil/water ratio), characterization of emulsions with high content of dispersed phase and their use for the preparation of porous scaffolds, analysis of interfacial tension between organic solvents and aqueous nanogel dispersions (including time-dependency), immobilization of nanogels at polymer/air interfaces and their characterization (AFM, SEM), calculation of Flory–Huggins parameters between nanogel network and solvents, and influence of temperature on emulsion phase diagrams and nanogel swelling (PDF)
