# Supplementary material for: Amphiphilic Nanogels as Versatile Stabilizers for Pickering Emulsions
Source: ACS Nano. 2024 Sep 4;18(37):25499–511. doi: 10.1021/acsnano.4c05143 (PMC11411724; doi:10.1021/acsnano.4c05143)
Supplement: Supplementary file 1 — nn4c05143_si_001.pdf [file nn4c05143_si_001.pdf]

ESI

Amphiphilic nanogels as versatile stabilizers for Pickering emulsions

Ruiguang Cui[1], Maret Ickler[2], Ante Markovina[1], Sidra Kanwal[1], Nicolas Vogel[2], Daniel Klinger[1, *]

[1] Freie Universität Berlin, Institute of Pharmacy, Königin-Luise-Str. 2-4, 14197 Berlin, Germany

[2] Friedrich-Alexander-Universität Erlangen-Nürnberg, Institute of Particle Technology 91058 Erlangen, Germany

*corresponding author: daniel.klinger@fu-berlin.de

Table of Contents

1. Materials 3
2. Instrumentation 3
3. Synthesis of PFPMA monomer 4
4. Synthesis of PPFPMA nanogels 4
5. Characterization of PPFPMA precursor particles 5
6. Synthesis of the nanogel library 5
7. FT-IR spectroscopy monitoring of functionalization of precursor particles 7
8. ^{1}H-NMR examination of copolymer composition 7
9. Angle-dependent dynamic light scattering 9
10. TEM investigation of particle size 9
11. Preparation of non-crosslinked amphiphilic copolymers 10
12. Contact angle of DODA copolymer films 10
13. Preparation of Pickering emulsions 11
14. Fluorescence microscopy images of Pickering emulsions 11
15. Influence of nanogel concentration on Tol/water Pickering emulsions 11
16. Droplet size and stability of toluene/water emulsion (DODA20, Tol25) 12
17. Phase diagram for toluene/water: Dependence on nanogel composition and toluene/water ratio 12
18. Emulsions with high content of dispersed phase 14
19. Determination of emulsion composition 14

20. Preparation of polystyrene particles from O/W PE 14
21. Preparation of polystyrene scaffolds from W/O emulsions with high content of dispersed phase 15
22. Interfacial tension between organic solvents and aqueous nanogel dispersions 16
23. Analysis of time-dependent interfacial tensions measurements 17
24. Immobilization of nanogels at polymer/air interfaces 20
25. AFM measurements of immobilized nanogels at the water/oil interface 21
26. Phase diagram for different oils: Dependence on nanogel composition and oil/water ratio 23
27. Diameter of nanogels in different solvents 25
28. Solubility of non-crosslinked amphiphilic copolymers 26
29. Calculation of Flory-Huggins parameters between nanogel network and solvents 27
30. Influence of temperature on the emulsion phase diagram: toluene/water 32
31. Examination of temperature-dependent nanogel swelling for DODA40 and DODA30. 33
32. Influence of temperature on the emulsion phase diagram: CyH/water 34
33. Influence of nanogel hydrophobicity on ROS/water emulsion at 60 °C 35
References 35

1. Materials

Acryloyl chloride, ethylene glycol dimethacrylate (EGDMA), 2-hydroxylpropyl amine (HPA), 2,6-Di-*tert*-butyl-4-methylphenol and 2,2-dimethoxy-2-phenylacetophenone (DMPA) were purchased from Sigma Aldrich. Acryloyl chloride was purified by vacuum distillation to remove the acryloyl chloride dimer. EGDMA was filtered through an Al_2O_3 column to remove the inhibitor before usage. 2,6-Lutidine, ammonium persulfate (APS), divinylbenzene (DVB), sodium dodecyl sulfate (SDS), and fluorescein sodium salt (NaFl) were purchased from Merck. Dodecylamine (DODA) was purchased from Alfa Aesar. Triethylamine (TEA) was purchased from abcr GmbH. Styrene was purchased from Acros and was used after washing with NaOH aqueous solution and subsequent distillation. Pentafluorophenol (PFP) and Nile red were purchased from Fluorochem. Polydimethylsiloxane (PDMS) elastomer kit (Sylgard 184) was purchased from Dow Corning. All chemicals without specific description were used as received. De-ionized (DI) water was obtained from LaboStar TWF systems (18.2 MΩ·cm, Siemens Ultrapure Water Systems). Glass slides (Sailing Boat, Cat. No. 7103 with single concave) were used for preparing fluorescence microscopy samples.

2. Instrumentation

The hydrodynamic diameter of nanogels was measured using dynamic light scattering (DLS) at different scattering angles and laser wavelength of 638.2 nm with Nicomp Nano Z3000 system. Transmission electron microscopy (TEM) images were obtained by the scanning transmission electron microscopy (STEM) mode of Hitachi FE-SEM SU8030 with the acceleration voltage of 30 kV and current of 10 μA. Scanning electron microscopy images were measured using the Hitachi FE-SEM SU8030 with acceleration voltage of 5 kV – 10 kV and current of 10 μA. Fourier-transform infrared spectroscopy (FT-IR) spectra were collected over the range of 4000 to 650 cm^{-1} by the Shimadzu IRSpirit (QATR-S) ATR FT-IR system. Fluorescence microscopy images were obtained by a Keyence BZ-X810 fluorescence microscope equipped with 4×/0.75 and 20×/0.75 objectives. Ultrasonic bath (Elmasonic S 30 H, ultrasonic frequency 37 kHz, 220-240 V, 280 W) and vortex mixer (neoLab Vortex Mixer 7-2020) at a speed of 3150 rpm were used to prepare Pickering emulsions. The interfacial tension (IFT) was measured by the pendant drop method with a Dataphysics OCA 20 analyzer. The contact angle was also measured with Dataphysics OCA 20 analyzer with the sessile drop method. Atomic force microscopy images were obtained with a JPK NanoWizard instrument in AC mode using a MikroMash NSC-18 / AL BS cantilever (resonance frequency 75 Hz, spring constant 2.8 N/m). The height profiles were measured after post processing the images with median and flattening fits in Gwyddion.

3. Synthesis of PFPMA monomer

The monomer pentafluorophenyl methacrylate (PFPMA) was synthesized according to a literature procedure[1]. Briefly, 60.2 g (327 mmol) of pentafluorophenol was dissolved in 250 mL of dichloromethane (DCM) in a 500 mL flask and then cooled in an ice bath. Then, 41.5 mL (38.2 g, 357 mmol, 1.09 times the molar amount of pentafluorophenol) of lutidine was added to the solution, and the mixture was allowed to cool for 10 minutes. Afterward, 40.5 mL (37.4 g, 357 mmol, 1.09 times the molar amount of pentafluorophenol) of methacryloyl chloride was added dropwise to the system over approximately 15 minutes. A pinkish-white salt formed quickly after the addition of methacryloyl chloride. The reaction mixture was stirred overnight and allowed to warm to room temperature. The product was purified by filtering off the salt, followed by washing the filtrate with water, saturated NH_4Cl (aq.), and saturated NaCl (aq.). The product was isolated by removing the solvent under reduced pressure in presence of 360 mg (1.6 mmol, 0.5 mol% to monomer) stabilizer 2,6-Di-*tert*-butyl-4-methylphenol, and purified by vacuum distillation to give PFPMA in a 78% yield (64 g). The successful synthesis of PFPMA was confirmed by NMR spectra (Figure S1) .^{1}H-NMR (400 MHz, Chloroform-d): δ = 6.47 – 6.42 (m, 1H, H_{cis}), 5.93 – 5.87 (m, 1H, H_{trans}), 2.11 – 2.05 (m, 3H, CH_3). ^{19}F-NMR (376 MHz, Chloroform-d): δ = -152.68 (d, J = 17.5, 2F, F_{ortho}), -158.10 (t, J = 21.6, 1F, F_{para}), -162.39 (dd, J = 22.3, 17.3, 2F, F_{meta}).

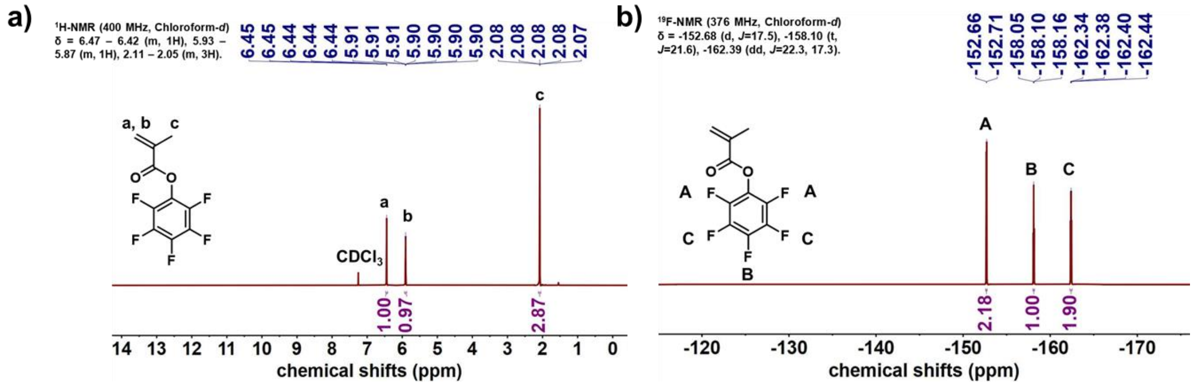

Figure S1. (a) ^{1}H-NMR spectrum and (b) ^{19}F-NMR spectrum confirm the successful synthesis of monomer PFPMA.

4. Synthesis of PPFPMA nanogels

PPFPMA nanogels were synthesized by emulsion polymerization according to modified literature procedures[2, 3]. In a typical recipe, 128.4 mg (0.445 mmol) surfactant SDS was added into a 250 mL flask. Then, a mixture of 21 g monomer PFPMA (83.3 mmol) and 153.4 µL (0.083 mmol) crosslinker EGDMA was added. After that, 210 mL water was added to adjust the

concentration of SDS to 2 mM. The flask was placed in a preheated oil bath (60 °C) and stirred under purging with nitrogen for 1.5 hours. Then, 2 mL of an aqueous APS solution with 126.6 mg/mL (1.1 mmol), which had been purged with nitrogen for 20 min in advance, was injected into the flask to initiate polymerization. The reaction was allowed to proceed under stirring for 5 days. Afterwards, the flask was opened to air and the PPFPMA particles were purified by centrifugation at 8000 rpm for 15 min, followed by washing with water for 4 times. Then the precursor particles were freeze-dried to give a white powder that can be used for functionalization. The yield was ~80% (17 g).

5. Characterization of PPFPMA precursor particles

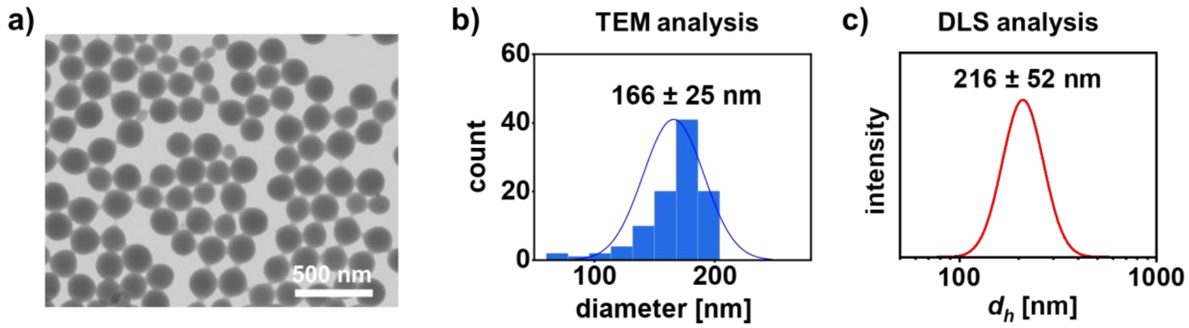

Figure S2. (a) TEM image, (b) statistical distribution curve of diameter calculated from TEM image and (c) statistical distribution curve of hydrodynamic diameter measured from DLS of PPFPMA precursor particles.

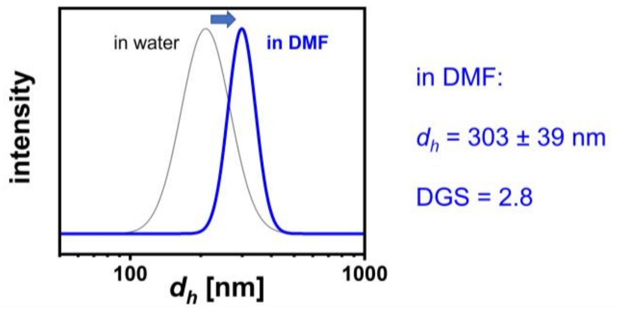

Figure S3. Compared to aqueous dispersions, PPFPMA precursor particles exhibit significant swelling in DMF, with a degree of swelling (DGS) of 2.8.

6. Synthesis of the nanogel library

The nanogels with different DODA contents were prepared by functionalizing the precursor PPFPMA nanoparticles according to the literature[4]. Briefly, 2 g (corresponding to 7.93 mmol PFPMA units) of the freeze-dried PPFPMA precursor particles were added in a 250 mL flask and dispersed in 150 mL DMF under ultrasonication for 30 min. A specific amount of dodecyl amine was dissolved in 25 mL DMF under ultrasonication at 40 °C and mixed with the

corresponding amount of 2-hydroxylpropyl amine. The amounts of dodecyl amine and 2-hydroxylpropyl amine for the different nanogels are summarized in Table S1. The mixture was then added into the PPFPMA dispersion, and the color changed to light green immediately. 3.310 mL TEA (23.64 mmol) as base were added. The reaction was stirred for 6 days at 60 °C. The resulting ANGs were purified by extensive dialysis against DMF and then against water. Note that the volume of the dispersion inside the dialysis tube expands twofold when changing from DMF to water. The purified DODA nanogel dispersion was concentrated by centrifugation at 10 k rpm for 2 hours and redispersion of the ANG in 40 mL water. The final concentration was gravimetrically determined by freeze-drying 1 mL of the dispersion. The yields of nanogels from DODA0 to DODA40 were 67% (0.76 g), 76% (0.93 g), 82% (1.07 g), 76% (1.06 g) and 76% (1.13 g), respectively. The hydrodynamic diameter (d_h) of nanogels in water was measured by diluting the concentrated samples with water to an approximate concentration of 0.1 mg/mL.

Table S1. Corresponding amount of HPA and DODA for DODA NGs synthesis.

	HPA			DODA	
	volume [mL]	mass [g]	n [mmol]	mass [g]	n [mmol]
DODA0	1.862	1.776	23.64	0	0
DODA10	1.676	1.598	21.28	0.438	2.36
DODA20	1.490	1.421	18.92	0.876	4.73
DODA30	1.303	1.243	16.55	1.312	7.08
DODA40	1.117	1.065	14.19	1.785	9.63

7. FT-IR spectroscopy monitoring of functionalization of precursor particles

Successful post-functionalization of precursor nanoparticles is demonstrated by the disappearance of the PPFPMA C=O ester band at 1776 cm^{-1} and the appearance of C=O amide band at 1657 cm^{-1} of the ANG products in FT-IR spectra.

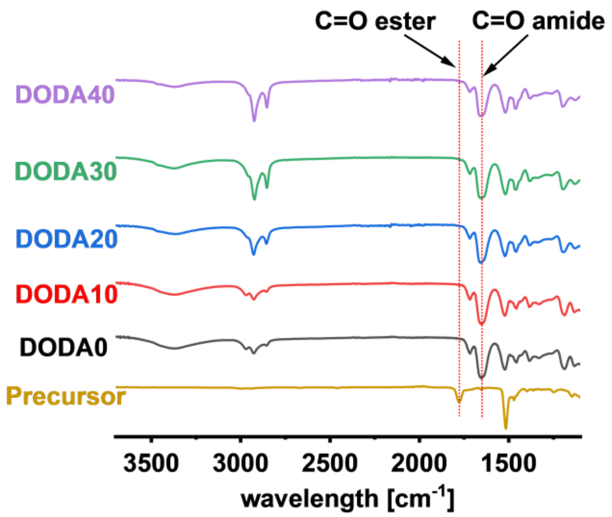

Figure S4. FT-IR spectra show the successful post-functionalization of precursor particles to DODA nanogels.

8. ^{1}H-NMR examination of copolymer composition

It is challenging to directly ascertain the chemical composition of nanogels using ^{1}H NMR spectroscopy due to their crosslinked structure, which leads to limited solubility in solvents. To enable NMR-based characterization, we synthesized non-crosslinked copolymer analogs of nanogels that are soluble in typical solvents (see below) for characterizing the chemical structure. Initially, the PPFPMA polymer was synthesized via Reversible Addition-Fragmentation chain Transfer (RAFT) polymerization, following a method documented in the literature[5]. Briefly, 10 g of PFPMA (40 mmol) was dissolved in 17 mL of 1,4-dioxane. Then, 0.185 g of 4-cyanopentanoic acid dithiobenzoate (CPADB) (0.66 mmol) and 21.7 mg of AIBN (0.132 mmol) were added to the mixture. The polymerization was initiated at 75 °C after being deoxygenated via freeze-vacuum-thaw cycles, and the reaction was allowed to proceed for 10 hours.

Subsequently, we applied the same post-functionalization protocol to create a series of DODA copolymers with varying dodecyl amine feeding ratios from 0 to 40%. After purifying the copolymers through extensive dialysis against DMF and water, approximately 20 mg of DODA copolymers were dissolved in their respective deuterated solvents (DODA0 in water, and DODA10 – DODA40 in DMSO) prior to ^{1}H NMR spectroscopy analysis.

In the ^{1}H NMR spectra analysis, the -C*H*(OH)CH_3 hydrogen in the HPA moiety (labelled with a red star in Figure S5a) served as the reference for normalization. The integrals of the other

hydrogen signals were summed, and after subtracting the contributions from the hydrogen atoms within the HPA moiety, this sum was used to calculate the molar ratio of the DODA moiety (as shown in Figure S5b).

GPC spectra were obtained for DODA0 – DODA40 copolymers, as shown in Figure S5c. The corresponding number-molecular weight and polydispersity of copolymers were summarized in Table S2. The GPC data show that all DODA copolymers show similar molecular weight, which is all smaller than 10 kDa and suitable for NMR analysis.

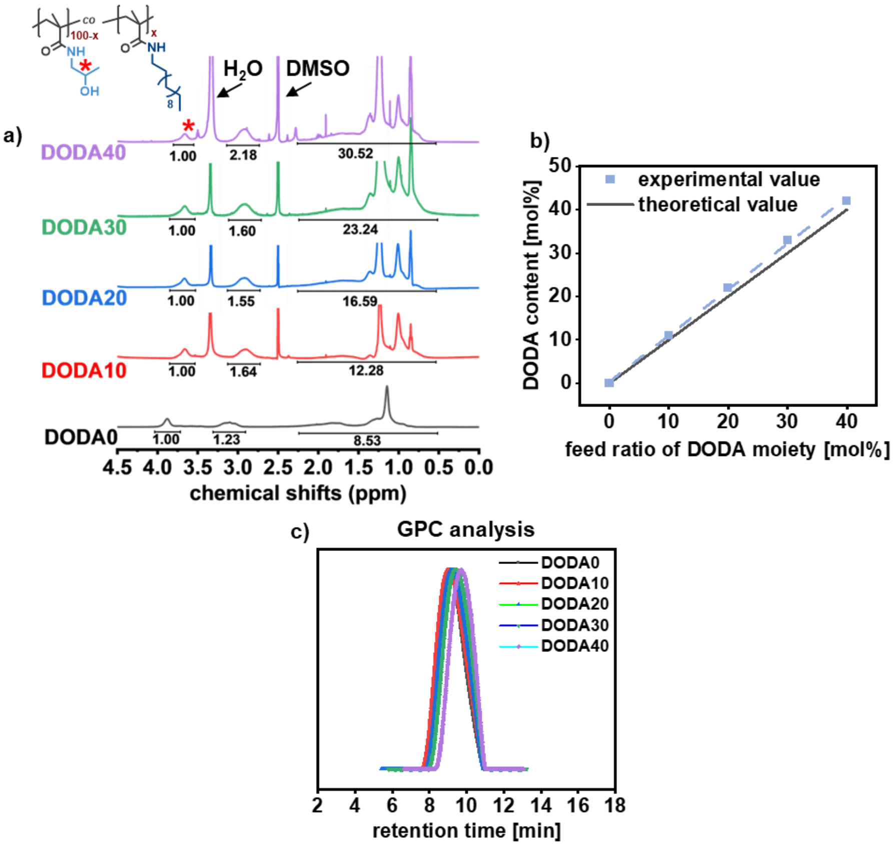

Figure S5. (a) ^{1}H-NMR spectra of DODA copolymers were used to calculate the incorporation of DODA moieties in the copolymer. (b) Experimental values of DODA content are consistent with theoretical values in DODA copolymers. (c) GPC traces of all copolymers.

Table S2. Number-average molecular weight and polydispersity of DODA copolymers.

	$M_{n,\ GPC}$ [g/mol]	*Đ*
DODA0	8.76 kDa	1.66
DODA10	8.61 kDa	1.71
DODA20	7.34 kDa	1.66
DODA30	6.97 kDa	1.77
DODA40	5.11 kDa	1.45

9. Angle-dependent dynamic light scattering

Hydrodynamic diameters for the nanogel dispersions in water were obtained from angle-dependent DLS measurements. For this, d_h was measured at the angles 70°, 80°, 90°, 100°, 110°. The obtained apparent diffusion coefficients D_{app} were plotted against the respective scattering vector q^2. Then a linear fit of the data was applied and extrapolated to $q^2 = 0$. The corresponding diffusion coefficient at $q^2 = 0$ was then used to calculate the respective hydrodynamic diameter. Figure S6 shows the plots of D_{app} vs q^2 for DODA0 – DODA40.

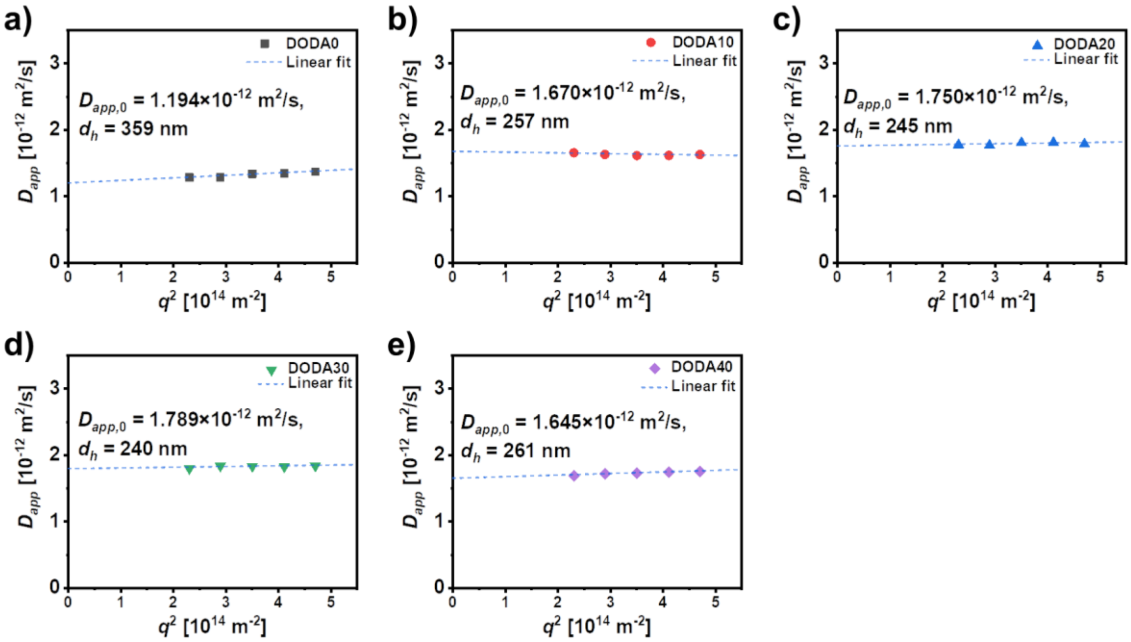

Figure S6. Z-average diffusion coefficients $D_{app,0}$ for DODA0 – DODA40 nanogels (a – e, respectively) were obtained by extrapolation to the y-intercept of the apparent diffusion coefficient D_{app} vs the square of the scattering vector q^2.

10. TEM investigation of particle size

The statistical diameter distribution was calculated using ImageJ. For this, the area of more than 100 particles was measured and converted to the respective diameter. As shown in Figure S7, all nanogels exhibit diameters similar to those of the precursor nanoparticles (166 ± 25 nm). DODA0 displays a larger size, likely due to enhanced swelling in water and greater deformation during drying on the TEM copper grid due to softer structure.

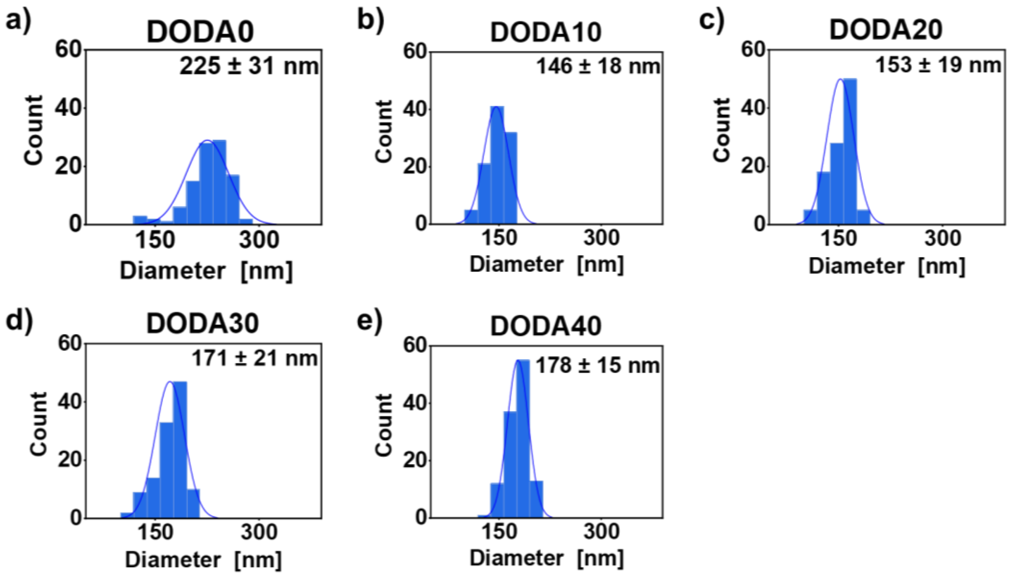

Figure S7. Diameter distribution curves of DODA0 – DODA40 nanogels (a – e, respectively) calculated from TEM images.

11. Preparation of non-crosslinked amphiphilic copolymers

Non-crosslinked amphiphilic copolymers were synthesized for contact angle measurements. Initially, PPFPMA non-crosslinked precursor polymers were prepared using the same protocol as for PPFPMA precursor particles, but without the addition of a crosslinker. This was followed by a post-functionalization process similar to that used for precursor polymers, resulting in the formation of non-crosslinked DODA polymers. The number-average molecular weight (M_n) of DODA0 – DODA40 copolymers is summarized in Table S3. The creation of new DODA copolymers, rather than utilizing those described in the ^{1}H-NMR characterization, was to match the copolymer composition as closely as possible with that of the nanogels, to get rid of factors such as molecular weight on the contact angle measurements.

Table S3. M_n and polydispersity of non-crosslinked DODA copolymers.

	$M_{n,\ GPC}$ [g/mol]	$Đ$
DODA0	76.40 kDa	1.51
DODA10	29.67 kDa	1.95
DODA20	34.25 kDa	1.87
DODA30	28.64 kDa	2.08
DODA40	81.76 kDa	2.71

12. Contact angle of DODA copolymer films

To assess the increasing hydrophobicity with the increasing hydrophobic DODA moieties of the DODOA copolymers, we measured the water contact angle on the polymer films. Typically, 50 mg/mL non-crosslinked DODA copolymer in DMF was prepared by sonication, followed by

centrifugation at 5 000 rpm for 10 min to remove any aggregates. Then 120 µL solution was evenly spread on a 2 cm x 2 cm silicon wafer, previously cleaned by ethanol with dust-free paper, and spin-coated at 3000 rpm for 1 minute to achieve a thin, uniform film. The silicon wafer was then dried in an oven at 70 °C for 10 hours to remove any residual solvent. The contact angle measurement was performed by depositing 5 µL of water onto the film and allowing the droplet to equilibrate. The contact angle was determined using the software provided by the instrument, employing the Young-Laplace fitting method, with the mean contact angle derived from five repeated measurements.

13. Preparation of Pickering emulsions

Nanogel dispersions with a concentration of 10 mg/mL (1 wt%) were prepared by diluting the concentrated nanogel samples with respective volume of an aqueous solution of NaFl (0.1 mg/mL). Organic oil phases contained 0.03 mg/mL of Nile red (toluene, DCM, 1-butanol, 1-decanol) or 0.5 mg/mL of anthracene (for cyclohexane as oil phase) due to poor solubility of Nile red in cyclohexane. To form Pickering emulsions, varying volumes of nanogel dispersion and organic solvent were combined to give a total volume of 4 mL in a glass vial (5 mL capacity). The mixture was then subjected to cycles of ultrasonication in ultrasonic bath for 1 minute followed by vortex mixing for 10 seconds, repeated 10 times. The ultrasonic bath was also capable of heating to 40 °C and 60 °C to create emulsions at these high temperatures.

14. Fluorescence microscopy images of Pickering emulsions

The Pickering emulsion was first diluted in the corresponding continuous phase in a 0.5 mL Eppendorf tube. Then 100 µL of the sample were placed on a glass slide with a concave dent in the middle. To reduce evaporation of liquid before observation under the fluorescence microscope, the samples were each covered with a cover slide and measured under a fluorescence microscope.

15. Influence of nanogel concentration on Tol/water Pickering emulsions

In a first experiment, an aqueous nanogel dispersion was emulsified with an excess amount of toluene, giving a system with 75 vol% of toluene (Tol-75). Testing different nanogel concentrations in the aqueous phase revealed that a minimum of 10 mg/mL was required to give stable emulsions for all samples from DODA0 - DODA40.

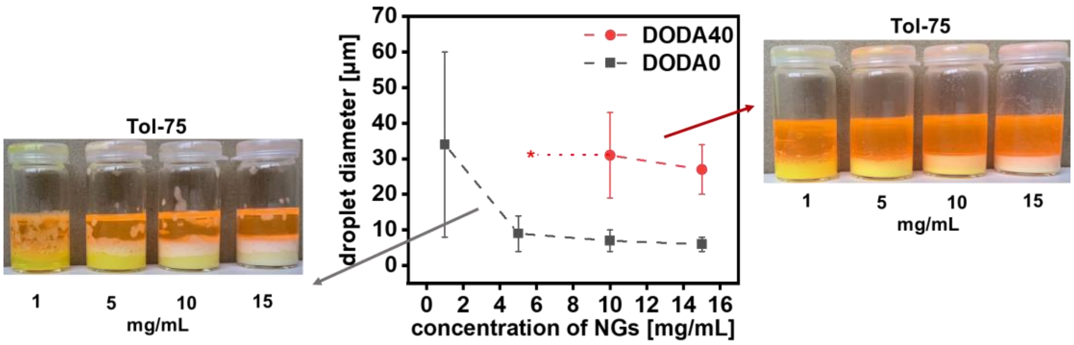

Figure S8. Droplet diameters of toluene/water (Tol-75) emulsions decrease with concentration of DODA0 and DODA40 nanogels respectively. *indicates emulsions are not stable prepared with 1 mg/mL and 5 mg/mL DODA40 nanogels.

16. Droplet size and stability of toluene/water emulsion (DODA20, Tol25)

To evaluate the stability of Pickering emulsions, we investigated the variation in the size of emulsion droplets over time. A vial containing the emulsion, specifically Tol-25 prepared with DODA20, was securely sealed using folded aluminum foil and a cap to minimize the evaporation of toluene. Microscopy images were analyzed using ImageJ, where the diameters of more than 1000 droplets were measured. The distribution of droplet diameters against the emulsion's duration was illustrated in a violin plot, as depicted in Figure S9. The consistent droplet diameters over 6 weeks demonstrate the high stability of Pickering emulsions stabilized by our nanogels.

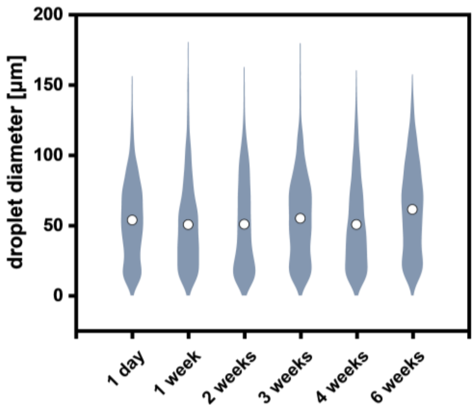

Figure S9. Consistent droplet diameters of emulsions over time show high stability of Pickering emulsions. The emulsions studied here are Tol-25 prepared with DODA20.

17. Phase diagram for toluene/water: Dependence on nanogel composition and toluene/water ratio

The oil phase was labeled with Nile red, which exhibits a red color under a fluorescence microscope, while the water phase was labeled with FITC-Na, exhibiting a green color under a fluorescence microscope. This labeling allows for easy determination of the dispersed and continuous phases based on color. It is noteworthy that in some samples, we observed a

mixture of single and double emulsions within the same sample. We define this kind of mixed emulsion, whether it consists of O/W + W/O/W or W/O + O/W/O configurations, as a complex emulsion, provided that the proportion of double emulsion droplets constitutes over 25% of the total droplet population.

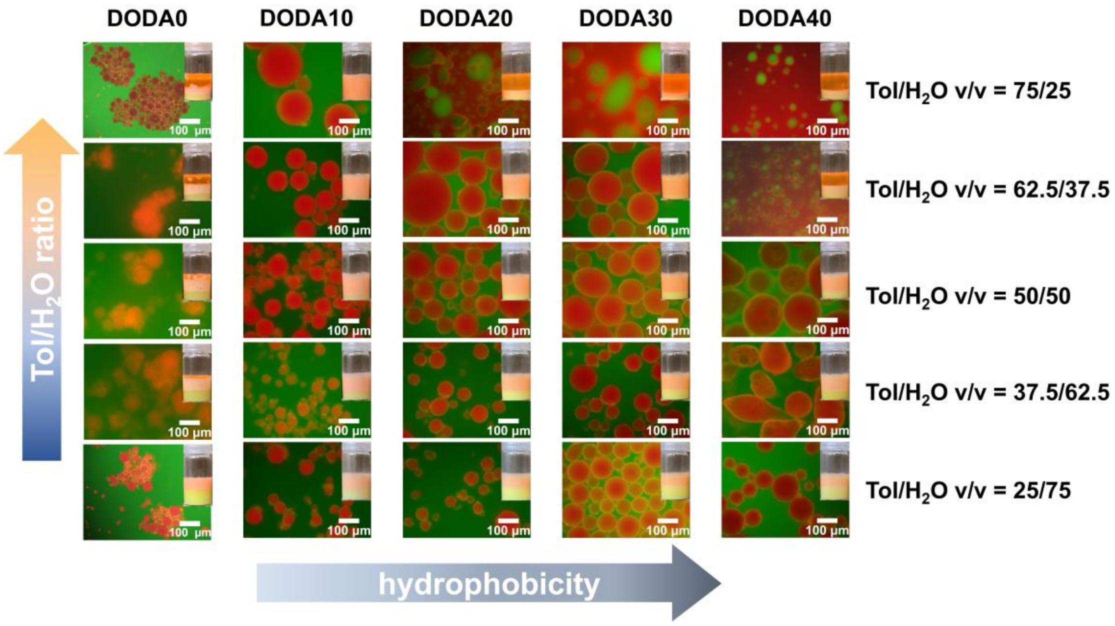

Figure S10. Fluorescence microscopy images and optical photographs of the vials containing the emulsions for the toluene/water system. Dependence on toluene/water ratio and nanogel hydrophobicity is examined.

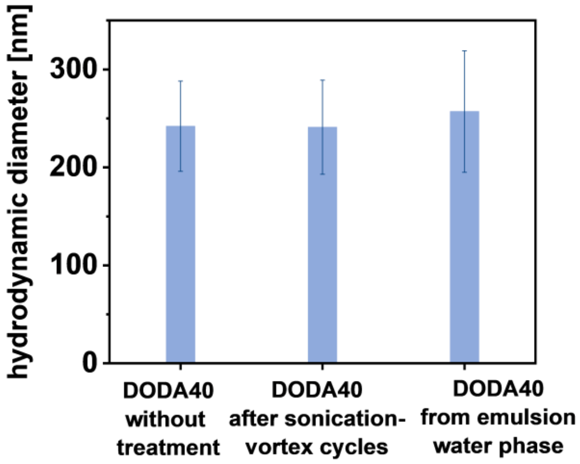

Figure S11. The size of nanogels (DODA40) does not change during the emulsification method (sonication-vortex cycles for 10 times), which indicates that nanogel aggregation does not influence the emulsification process.

18. Emulsions with high content of dispersed phase

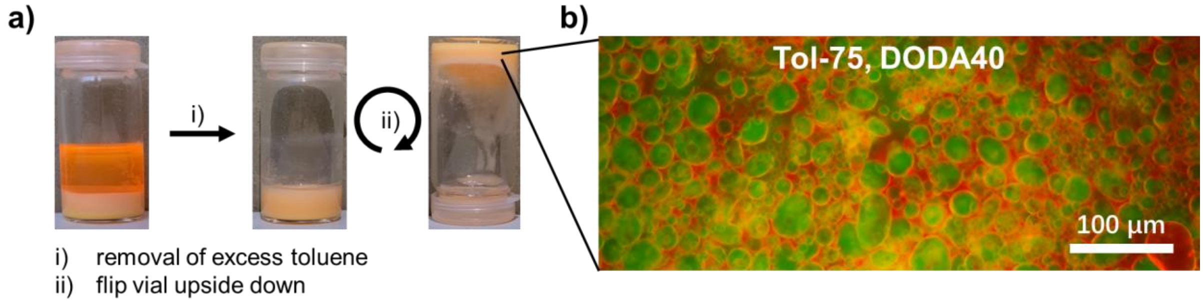

Figure S12. Tol-75 Pickering emulsion with high content of dispersed phase prepared with DODA40. (a) Following the preparation of the Tol-75 emulsion, excess toluene in the supernatant was removed. Subsequently, when the glass vial was inverted, a viscous gel was observed, indicative of the viscous nature characteristic emulsions high content of dispersed phase. (b) A fluorescence microscopy image of the undiluted emulsion reveals concentrated water droplets, typical of emulsions with high content of dispersed phase.

19. Determination of emulsion composition

To determine the composition of the emulsion, our analysis focused exclusively on the emulsion part, intentionally excluding any excess supernatant or sediment. To calculate the volume fraction of the dispersed phase in the emulsion, we divided the volume of the dispersed phase by the total volume of the emulsion part. For a specific sample, the volume of dispersed phase was determined by subtracting the volume of any excess dispersed phase from the feed value. For instance, in the case of the Tol-25 emulsion prepared with DODA40, the known volume of the dispersed phase (toluene) was 1 mL. Given that the volume of the emulsion part was 1.88 mL, the volume fraction of the dispersed phase within the emulsion part was calculated to be 1 mL / 1.88 mL = 53%.

Table S4. Determined volume fractions of dispersed phase in the emulsion part.

	DODA0*	DODA10	DODA20	DODA30	DODA40
Tol-75	91% ± 8%	75%	73% ± 2%	79% ± 2%	76% ± 2%
Tol-62.5	73% ± 2%	72% ± 1%	71% ± 0%	73% ± 0%	78% ± 3%
Tol-50	71% ± 5%	61% ± 3%	65% ± 2%	69% ± 2%	70% ± 1%
Tol-37.5	60% ± 3%	55% ± 1%	58% ± 1%	63% ± 3%	56% ± 1%
Tol-25	56% ± 1%	37% ± 2%	48% ± 1%	52% ± 1%	53% ± 2%

20. Preparation of polystyrene particles from O/W PE

To observe the nanogels at the surface of oil droplets, we immobilized the nanogels by polymerizing the oil phase with styrene as toluene analogue. 1 mL monomer oil phase, consisting of a mixture of 950 µL styrene, 50 µL divinyl benzene, and 20 mg AIBN, was added

to a 5 mL glass vial containing 3 mL of a 10 mg/mL DODA20 nanogel dispersion. The mixture was purged with argon for 5 minutes, then subjected to cycles of ultrasonication in an ultrasonic bath for 1 minute followed by vortex mixing for 10 seconds, repeated 10 times. Subsequently, the emulsion was placed in a 70 °C oil bath to initiate polymerization and was left overnight to react. The formed polystyrene microparticles were centrifuged at 3 000 rpm for 10 min and washed with water afterwards for 5 rounds. The dispersion of polystyrene particles was dried on a silicon wafer before gold sputtering for SEM measurements.

21. Preparation of polystyrene scaffolds from W/O emulsions with high content of dispersed phase

In a 5 mL glass vial, a Tol-75 emulsion was prepared by mixing 3 mL toluene and 1 mL DODA40 nanogel dispersion (10 mg/mL) through ultrasonication and vortex mixing. After allowing the emulsion part to settle for 3 hours, the excess supernatant toluene was carefully decanted. The vial was then filled with a monomer mixture comprising 2.75 mL of styrene, 150 µL of divinylbenzene (DVB), and 60 mg of azobisisobutyronitrile (AIBN), which had been purged with argon for 5 minutes to remove oxygen. This emulsion + monomer mixture was vortexed for 20 seconds and then left undisturbed for 3 hours to ensure sedimentation of emulsion part, before initiating polymerization by placing the vial in a 70 °C oil bath and leaving overnight to react. The formed porous material was carefully removed from the vial and freeze-dried to remove any residual solvents. For further structural analysis, the dried sample was immersed in liquid nitrogen for 10 minutes, and then fractured by hand to expose the interior. For examination via SEM, the intersection was sputtered with gold beforehand.

From SEM images (Figure S13), we observe a layer of nanogels on the inner surface of the pores, originating from the W/O emulsions' water droplets. Interestingly, the nanogels exhibit a non-close packed structure on the droplet surface, different from the close packing seen in Figure 2c of the O/W emulsions.

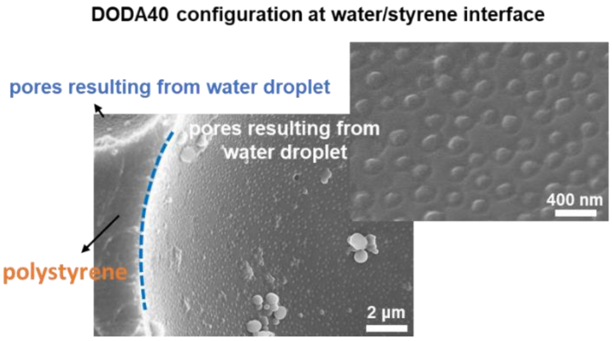

Figure S13. In the polystyrene porous material, high magnification SEM images show a non-close packed structure of DODA40 nanogels at the inner pore surface. The pores were originated from the water phase in W/O emulsions stabilized by DODA40 nanogels.

22. Interfacial tension between organic solvents and aqueous nanogel dispersions

Before the measurements, all samples were extensively purified. For this, freeze-dried DODA10, DODA20, DODA30 samples were redispersed in DMF (10 mg/mL) and washed with DMF via centrifugation (10k rpm, 2 hours) and redispersion for 3 times. Finally, the samples were extensively dialyzed against water. For DODA40, 1-butanol was used for redispersion and washing in a similar procedure. Then DODA40 was first dialyzed against DMF to remove 1-butanol and then against water to remove any organic solvents. All samples were concentrated by centrifugation and redispersion in smaller amounts of water. The concentration of all different nanogels in aqueous dispersion was gravimetrically determined by weighing a freeze-dried aliquot of the sample. Finally, the concentration was adjusted to 10 mg/mL by dilution with water. The interfacial tension (IFT) was measured by the pendant drop method. For this, a 50 μL drop of the aqueous nanogel dispersion (10 mg/mL) was suspended in the toluene (HPLC grade) in a quartz cuvette. IFT data were recorded at a constant rate of 10 points/min to study the kinetics of IFT decrease.

We hypothesize that interphase transfer of hydrophobic nanogels from the water phase to the oil phase is unlikely because of the large interfacial desorption energy (the particles would need to cross the interface to partition into the toluene phase). Therefore, we only consider adsorption processes from the water phase in our analysis. We assess this hypothesis by investigating nanogel partitioning into a toluene supernatant after forming a water-in-toluene emulsion with 75/25 (vol/vol) toluene/water and DODA40 nanogels. In this system, the nanogels were initially dispersed in the water phase. After emulsification, we observed a separation of the system into the emulsion fraction and a transparent supernatant of excess toluene. We measured the light scattering intensity of the supernatant using dynamic light scattering (DLS). We compared this scattering intensity to that of a 0.5 mg/mL DODA40 nanogel dispersion in toluene as a positive control, and a pure toluene sample (8 kHz) as a negative control. As can be seen in Figure S14, the scattering intensity of the toluene supernatant (8 kHz) is comparable to that of pure toluene, but around 20 times lower than that of the 0.5 mg/mL nanogel dispersion. These results suggest that there is negligible interphase transfer of hydrophobic nanogels from the water phase to the oil phase.

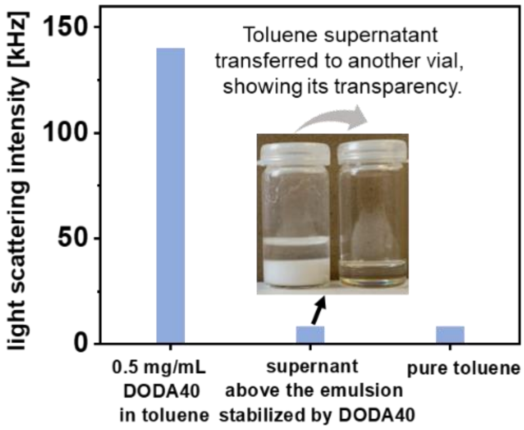

Figure S14. For water-in-toluene emulsions (DODA40, 75/25 (vol/vol) toluene/water), the supernatant of excess toluene appears transparent and shows similar low scattering intensity as pure toluene, which strongly suggests that there is no interphase transfer of hydrophobic nanogels from water phase to oil phase.

23. Analysis of time-dependent interfacial tensions measurements

From the kinetic curves of *IFT* vs. *t*, the different processes can be extracted as follows:

For the first process (P1), the diffusion coefficient *D* is connected to the interfacial tension γ via equation (1).[6, 7]

(1)
$$\gamma_0 - \gamma_t = 2RT\sqrt{\frac{D}{\pi}}C_{bulk}\sqrt{t}$$

Here, γ_0 and γ_t describe the interfacial tension of toluene/water at the beginning and at time *t*, respectively; R is the gas constant; *T* is temperature; *D* is the diffusion coefficient of nanogels, which indicates the diffusion rate of nanogels to the interface; C_{bulk} is the bulk concentration of nanogels. By plotting (γ_t - γ_0) vs. $t^{1/2}$, the short initial stages can be fitted with a linear relationship according to equation (2).

(2)
$$\gamma_0 - \gamma_t = k_1 \cdot t^{1/2}$$

The diffusion coefficient *D* can be calculated from the slope k_1 of the fit, where the nanogel concentration C_{bulk} [mol/L] is calculated from the mass of particles, their volume from TEM, and an estimated polymer density of 1 g/cm^3. The obtained values are summarized in Table S5.

Table S5. Key parameters of the first stage of interfacial tension kinetics.

	Diameter[(1)] [nm]	m_0[(2)] [g]	c[(3)] [mg/mL]	C_{bulk} [10^{-6} M]	Slope k_1	D[(4)]	D_{rel}[(5)]
DODA0	225	5.96×10^{-15}	10	0.00279	1.40	3.37×10^{-2}	1
DODA10	146	1.63×10^{-15}	10	0.0102	0.10	1.31×10^{-5}	3.88×10^{-4}
DODA20	153	1.87×10^{-15}	10	0.00886	0.090	1.33×10^{-5}	3.94×10^{-4}
DODA30	171	2.62×10^{-15}	10	0.00635	0.13	5.79×10^{-5}	1.72×10^{-3}
DODA40	178	2.95×10^{-15}	10	0.00563	0.19	1.50×10^{-4}	4.45×10^{-3}

(1) calculated from TEM images.
(2) mass of a single nanogel. The density of nanogels was set as 1 g/cm^3.
(3) mass concentration of nanogels in water.
(4) diffusion coefficient of the 1st stage of interfacial tension kinetics.
(5) relative diffusion coefficient (w.r.t. DODA0).

The second process (P2), of interfacial tension kinetics can be described by an exponential relaxation due to first-order kinetics according to equation (3)[6]:

(3)
$$\frac{d\gamma_t}{dt} = -k_2(\gamma_t - \gamma_\infty)$$

This leads to equation (4):

(4)
$$\ln(\gamma_t - \gamma_\infty) = -k_2 t + \ln(\gamma_0 - \gamma_\infty)$$

By plotting ln(γ_t - γ_∞) vs. t the data can be linearized and fitted respectively to obtain k_2. For a first-order process, the slope k_2 can be used to calculate the half-life, $t_{1/2}$, of the second process P2 according to equation (5).

(5)
$$t_{1/2} = \frac{\ln 2}{k_2}$$

We hypothesize that the interfacial adsorption and rearrangement kinetics of nanogels, shown in Figure 3, influence the efficiency with which they can stabilize emulsions. To examine this hypothesis, we used toluene/water emulsions (50/50 vol/vol) as model systems and estimated the interfacial adsorption efficiency via two complementary methods. First, we measured the fraction of interfacially adsorbed nanogels indirectly by gravimetrically determining the amount of non-adsorbed nanogels. The mass of nanogels before emulsification was 20 mg for all emulsion samples. After emulsification and creaming of the emulsions, we extracted 300 μL of the lower water phase (which did not contain any visible toluene droplets) and freeze-dried the solution to gravimetrically measure the remaining nanogels. Since we showed that nanogels do not partition into the toluene phase (see Figure S14), we can determine the fraction of interfacially adsorbed nanogels from the total amount of non-adsorbed microgel mass in the

total volume of the dispersed water phase (2 mL), as shown in Figure S 15. We found that with increasing hydrophobicity, the adsorption efficiency of the nanogels first decreases to around 25% for DODA20 and DODA30, then increases to around 50% for DODA40. This trend qualitatively agrees with the nanogel adsorption and rearrangement kinetics at the interface, which shows faster adsorption for both hydrophilic DODA0 and hydrophobic DODA40 compared to the samples with intermediate hydrophobicities (Figure 3 in the main text).

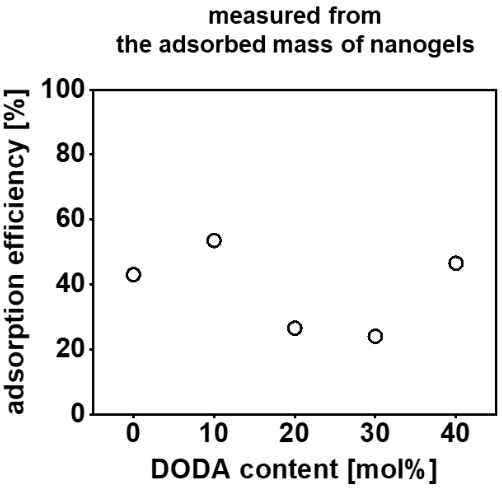

Figure S15. The adsorption efficiency of different nanogels at the toluene/water interfaces, as measured from the adsorbed mass of nanogels from the 50/50 (vol/vol) toluene/water emulsions.

In addition, we mathematically estimated the adsorption efficiency of nanogels by considering the total emulsified toluene volume, the droplet size (which gives its surface area) and the nanogel size at the interface. Assuming a close-packed arrangement of nanogels at the interface, we can use the total available surface area to estimate how many nanogels are adsorbed at an interface as follows:

$$m_{adsorption} = \frac{(V_{emulsified} / V_{0droplet}) \cdot A_{0droplet}}{A_{0NG}} \cdot m_{0NG}$$

where $V_{emulsified}$ is the experimentally determined emulsified volume of toluene; $V_{0droplet}$ is the volume per emulsion droplet and $A_{0droplet}$ is the surface area per emulsion droplet (calculated from Figure S26); A_{0NG} is the area per nanogel occupies at the toluene/water interface (calculated from Figure S21); and m_{0NG} is the mass per nanogel (Table S5). Finally, the adsorption efficiency can be estimated given the total mass of nanogels (20 mg).

The results of this simplified estimation support the trend of lower adsorption efficiency of nanogels with intermediate hydrophobicity compared to other nanogels (Figure S16).

Note that quantitative differences between the values in Figure S15 and Figure S16 could be attributed to errors in the assumptions of monodispersed emulsion droplets and the dense, homogeneous packing of nanogels at the interfaces, the estimation of the mass per nanogel etc. However, we observed a similar trend from both complementary methods: nanogels with intermediate hydrophobicity adsorb less than their more hydrophilic or hydrophobic counterparts, which also agrees to the adsorption and rearrangement kinetics study (Figure 3).

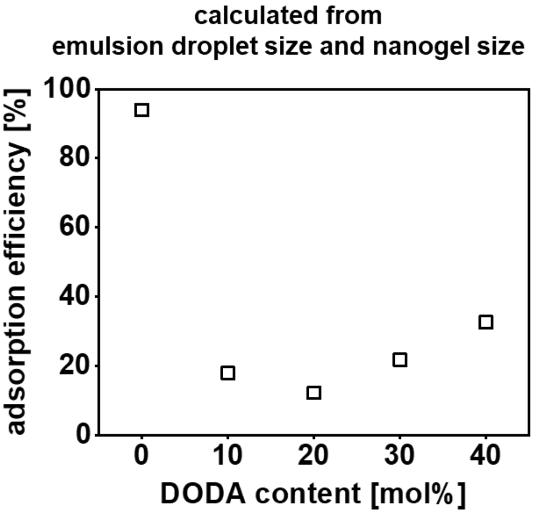

Figure S16. The adsorption efficiency of different nanogels at the toluene/water interfaces, as mathematically estimated from the emulsion droplet size and the nanogel size at the interfaces. The 50/50 (vol/vol) toluene/water emulsions are used for the calculation here.

24. Immobilization of nanogels at polymer/air interfaces

To observe the nanogel positioning at the oil/water interface, we employed the gel-trapping technique to immobilize the nanogels within a gel matrix[8]. First, in a 5 mL glass vial, 40 mg agarose was dissolved in 2 mL water at 90 °C in an oil bath under continuous stirring to dissolve the agarose achieving a concentration of 20 mg/mL. Upon complete dissolution, the stirring bar was removed. Second, 1 mL of toluene, preheated to 90 °C, was gently layered atop the agarose solution. Third, the vial was transferred to a 60 °C water bath to maintain the agarose in solution form and left undisturbed for 30 min to achieve thermal equilibrium. Fourth, a nanogel dispersion in ethanol was prepared by adding 200 µL of a 1 mg/mL aqueous nanogel solution to 200 µL of ethanol, reaching a final concentration of 500 µg/mL. A volume of 100 µL of this dispersion was then gently introduced to the toluene-water interface within the vial using a micropipette. The system was allowed to equilibrate for 3 hours to facilitate the equilibration of nanogels at the interface. Following this period, the vial was set aside to cool to room temperature, triggering gelation of the agarose, a process that took approximately 1-2 hours.

After gel formation, the toluene was carefully decanted from the vial, which was subsequently filled with roughly 5 mm thick layer of PDMS. The PDMS was prepared as follows: 2 g Polydimethylsiloxane (PDMS) base and 0.2 g corresponding curing agent were blended in a centrifuge tube, which inevitably introduced bubbles. To eliminate these bubbles, the mixture was centrifuged at 5000 rpm for 5 minutes. The PDMS gel mixture transferred atop agarose gel was then left to stand for 48 hours in an open vial within a fume hood to solidify. Subsequently, the PDMS layer was extracted from the gel phase using tweezers. Residual agarose was removed by immersing the PDMS gel in water (90 °C) for 10 minutes and rinsing with water, repeated three times. The PDMS film was then air-dried in the fume hood. Prior to scanning electron microscopy (SEM) examination, the PDMS gel was coated with gold.

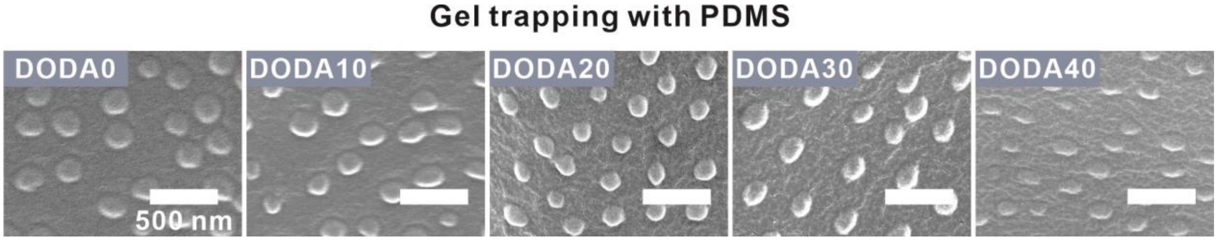

Figure S17. SEM images of DODA0 – DODA40 nanogels on the PDMS gel indicate different protrusion depths of nanogels to the oil phase. DODA40 obviously protrudes more in the PDMS gel than other nanogels.

We also verified the positioning of nanogels at the toluene/water interface by a similar gel-trapping technique but with solidification of styrene instead. Firstly, we utilized the same protocol to equilibrate nanogels at the toluene/water interface as described above for PDMS gel trapping technique, i.e., adding toluene atop an agarose aqueous solution, transferring to a warm water bath, afterwards spreading nanogel dispersion to the toluene/water interface and cooling to room temperature to let agarose form gel. Secondly, the toluene was carefully decanted from the vial, which was subsequently filled with roughly 5 mm thick layer of mixture of 1 mL styrene with photoinitiator 2,2-Dimethoxy-2-phenylacetophenone (DMPA). This mixture had been deoxygenated by argon bubbling for 5 minutes. Photopolymerization of the styrene was carried out under 365 nm UV light at room temperature, ensuring that the agarose remained in its gelled state. Thirdly, the polystyrene layer was extracted from the gel phase using tweezers and washed in the same way as in the PDMS gel trapping technique and sputtered gold before measuring SEM.

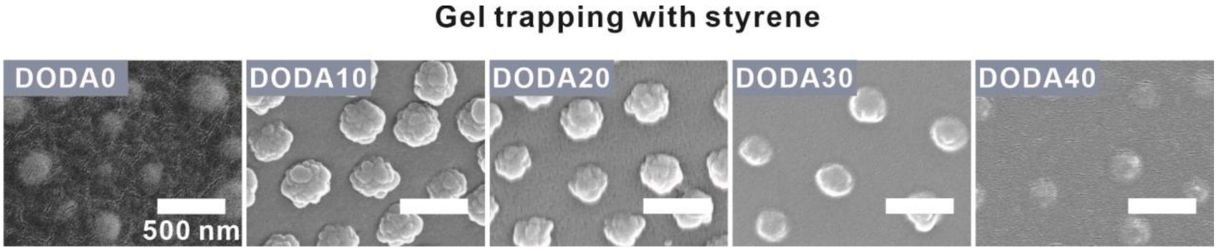

Figure S18. SEM images of DODA0 – DODA40 nanogels on a polystyrene substrate illustrate the varying depths of nanogel protrusion into the oil phase. It is evident that DODA40 nanogels protrude more into the substrate compared to other nanogels, while DODA10 – DODA30 nanogels are more extended outwards. Notably, for DODA10 and DODA20, the presence of bumpy structures on the particle surface is observed, which may be attributed to swelling of the nanogel networks with styrene and subsequent polymerization within, thus leading to the formation of composite nanoparticles.

25. AFM measurements of immobilized nanogels at the water/oil interface

To quantitatively evaluate the protrusion depth of nanogels outwards and inwards the PDMS gel, which indicates the protrusion to water side and oil side, respectively, atomic force microscopy (AFM) was employed. AFM measurements were taken of the nanogels on the PDMS gel surface and of the cavities formed after nanogel removal – accomplished by mechanical rubbing for DODA0 and by an adhesive tape for DODA10 – DODA40. The AFM images capturing the water-side nanogels are presented in Figure S19, whereas those on the oil side are depicted in Figure S20 Subsequently, the profiles of both the nanogels and the resultant holes were analyzed using Gwyddion software. The resulting data, shown in Figure S21 reveals the varying extents of deformation experienced by different nanogels.

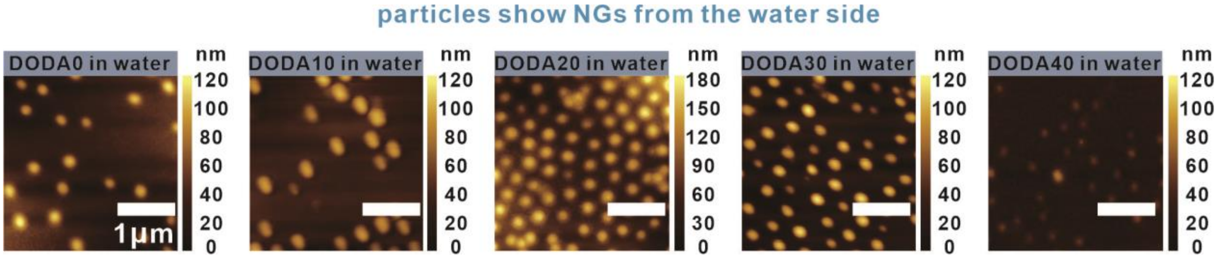

Figure S19. AFM images show nanogels (from DODA0 to DODA40) on PDMS gel substrate, which indicate the original protrusion depth of nanogels in the water phase.

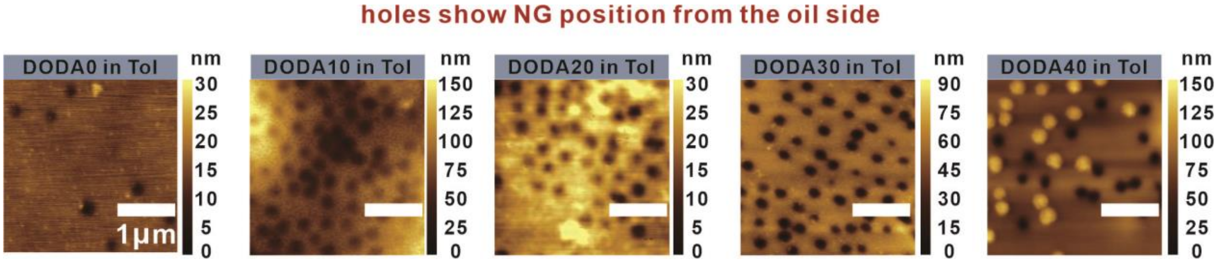

Figure S20. AFM images show holes left by removal of nanogels (from DODA0 to DODA40) from PDMS gel substrate, which indicate the original protrusion depth of nanogels in the toluene phase.

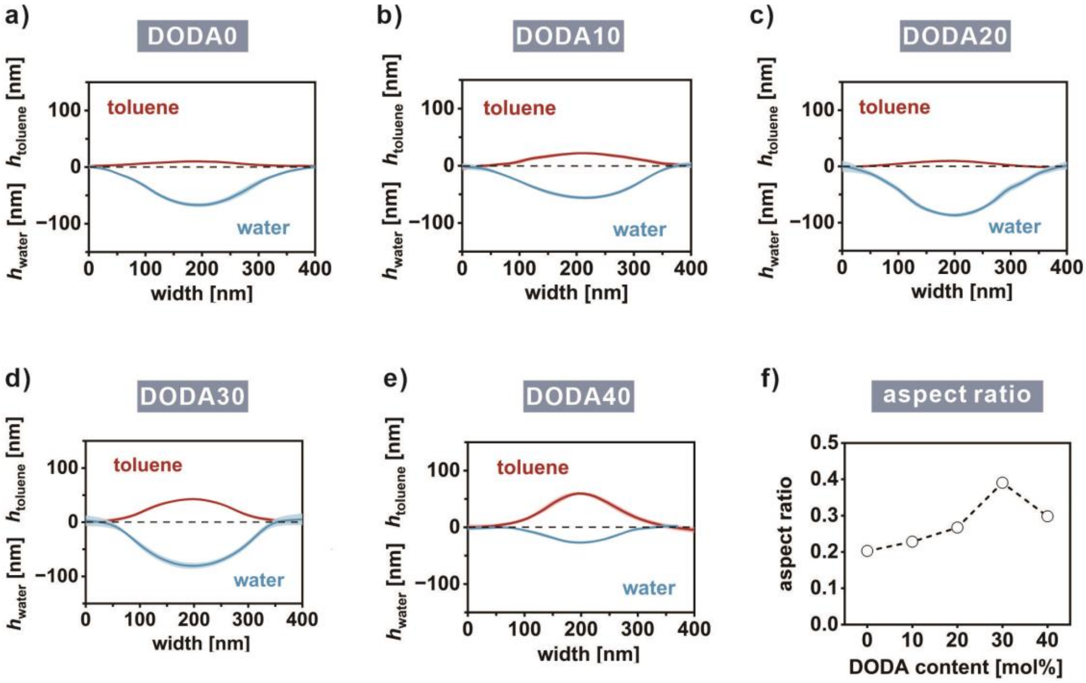

Figure S21. Variation in deformation at the toluene/water interface among different DODA nanogels. (a – e) Profiles of nanogels ranging from DODA0 to DODA40, respectively, in toluene and water, as determined from AFM images. (f) Aspect ratio derived from the formula (height in toluene + height in water) / length, indicating that DODA30 exhibits the least extent of deformation.

26. Phase diagram for different oils: Dependence on nanogel composition and oil/water ratio

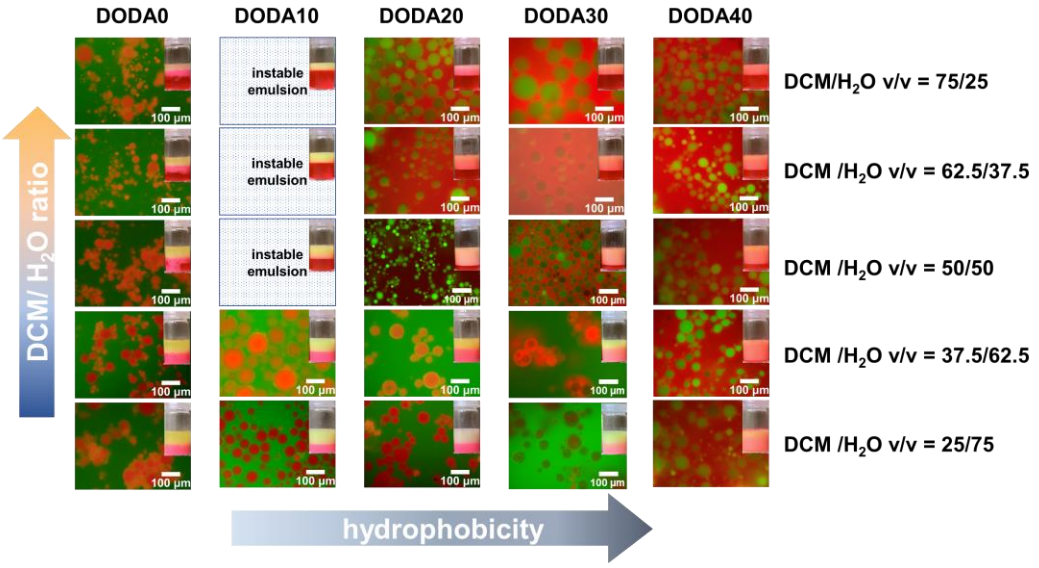

Figure S22. Fluorescence microscopy images and optical photographs of the vials containing the emulsions for the DCM/water system. Dependence on toluene/water ratio and nanogel hydrophobicity is examined.

Note that for DCM-50 to DCM-75, DODA10 did not form stable emulsions. Since DODA0 can form O/W emulsions, and DODA20 – DODA40 lead to W/O emulsions, it is reasonable to assume that DODA10 resides at the middle of the DCM/water interface. We suggest that such intermediate amphiphilic nanogels lack the efficiency to form emulsions, possibly due to the slower kinetics of the interfacial tension decrease.

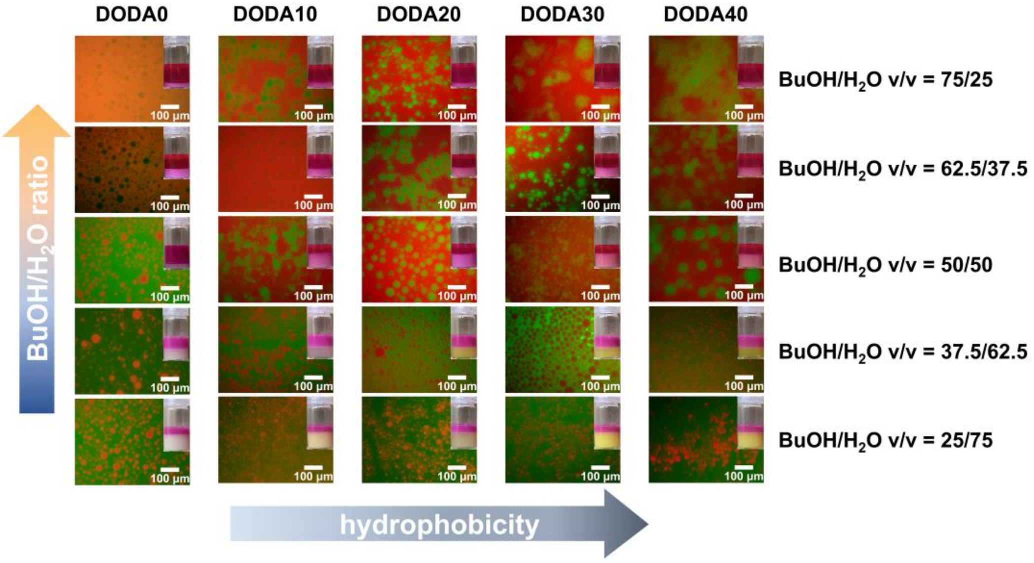

Figure S23. Fluorescence microscopy images and optical photographs of the vials containing the emulsions for the BuOH/water system. Dependence on BuOH/water ratio and nanogel hydrophobicity is examined.

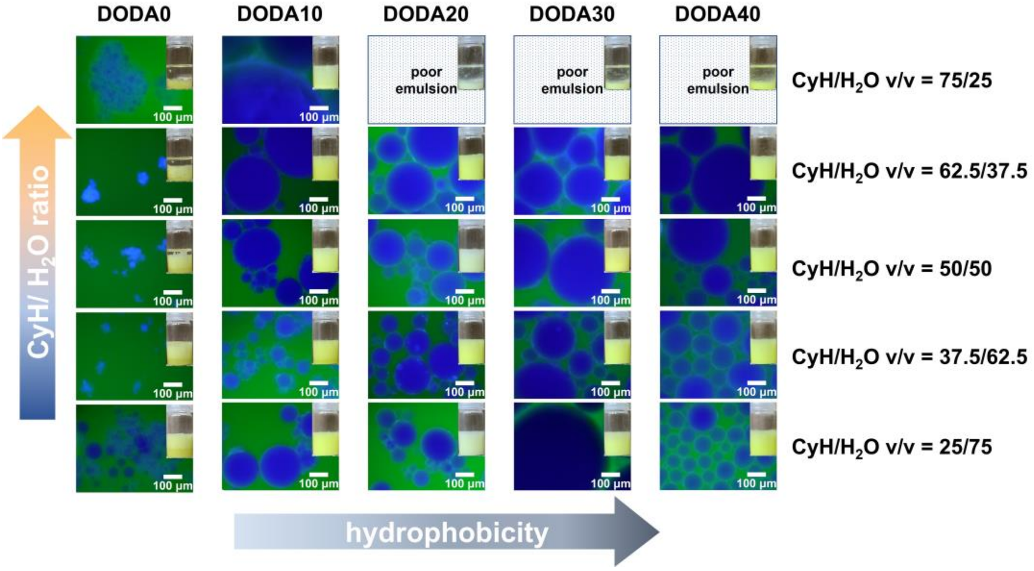

Figure S24. Fluorescence microscopy images and optical photographs of the vials containing the emulsions for the cyclohexane/water system. Dependence on CyH/water ratio and nanogel hydrophobicity is examined.

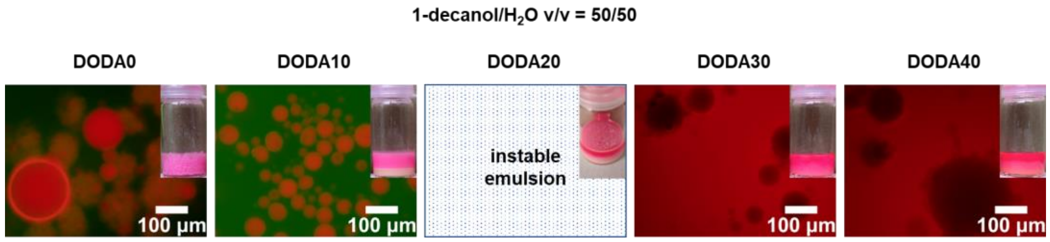

Figure S25. Fluorescence microscopy images and optical photographs of the vials containing the emulsions for the 1-decanol/water system at a ratio of 1-DecOH/water of 50/50 v/v. Dependence on nanogel hydrophobicity is examined.

Emulsion droplet size vs. oil type, nanogel hydrophobicity and emulsion type.

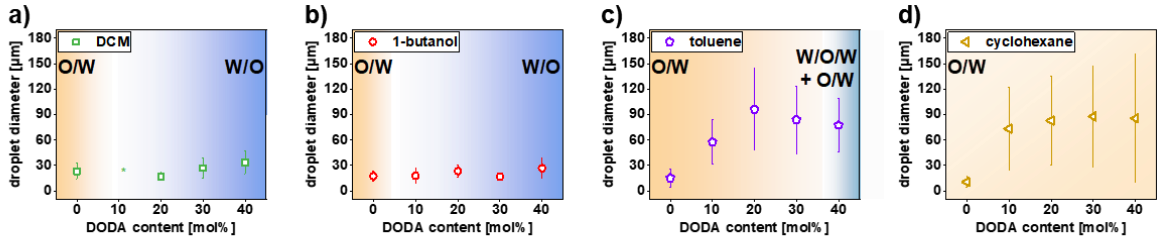

Figure S26. Emulsion droplet size of various emulsion systems prepared with 50/50 (vol/vol ratio) of oil/water as a function of DODA content: a) DCM/water, b) 1-butanol/water, c) toluene/water, and d) cyclohexane/water. (* for DCM/water with DODA10 there was no emulsion formed.)

We further studied the influence of emulsification ability of different nanogels from the viewpoint of the emulsion droplet size of various emulsion systems, all with the volume ratio of oil/water of 50/50 (Figure S26). Firstly, we noticed that the droplet size changes with oil type. Here the droplet size of DCM/water (< 30 μm) and 1-butanol/water (< 30 μm) systems are smaller than the droplet size of toluene/water (> 60 μm) and cyclohexane/water (> 60 μm) emulsions. The smaller size of DCM/water and 1-butanol/water emulsion droplets is may result from to the lower interfacial tension of DCM/water (28.3 mN/m)[9] and 1-butanol/water (1.8 mN/m)[9], compared with toluene/water (35.8 mN/m)[10] and cyclohexane/water (50.0 mN/m).[9]

Secondly, we note that the different oils show different evolutions of droplet sizes as a function of nanogel hydrophobicity. For polar oils (DCM, BuOH), the droplet size seems independent of the DODA content. In contrast, for non-polar oils such as toluene, DODA0 nanogels the resulting emulsion droplet size is smallest, indicating that DODA0 is the most efficient stabilizer. This smaller droplet size agrees with the fastest adsorption kinetics and lowest interfacial tension of toluene/water with DODA0 nanogels, as discussed in Figure 3. In addition, though the droplet size distribution of toluene/water emulsions is broad for DODA10 to DODA40, a slight trend is noticeable. From DODA0 to DODA20 the droplet size increases, while it decreases again to DODA40. The larger droplet sizes for DODA20-DODA30 compared to both DODA0 and DODA40 may be attributed to the slower decrease in interfacial tension for these nanogels, as can be seen in Figure 3. For cyclohexane/water emulsions, DODA10 to DODA40 stabilized emulsions show quite large droplets, which indicates the lower efficiency of DODA10 to DODA40 nanogels to emulsify cyclohexane/water system.

Thirdly, for both DCM/water and 1-butanol/water systems, the droplet size of O/W and W/O is similar, which suggests that the emulsion droplet size is not significantly affected by the emulsion type. This data suggests that the droplet size of Pickering emulsions is more correlated to the oil type and the nanogels' intrinsic properties, less to the emulsion type.

27. Diameter of nanogels in different solvents

The hydrodynamic diameter (d_h) of nanogels in water was measured by diluting the concentrated samples with water to an approximate concentration of 0.1 mg/mL. To determine d_h of nanogels in 1-butanol, the aqueous nanogel dispersions were first freeze-dried and the powder was then dispersed in 5 mL DMF (1 mg/mL). The DMF dispersion was dialyzed against 200 mL DMF for ~2 h. Then, 1/10 of the outer solvent was removed and replaced by the same amount of 1-butanol. After ~2 h, this procedure was repeated. In total this was done 10 times. Afterwards, the sample was dialyzed against pure 1-butanol for 3 days with changing solvents 5 times a day before the diameter was determined via DLS. For determining d_h in toluene, the nanogel dispersion in 1-butanol was subjected to a similar procedure by gradually replacing

1-butanol with toluene before DLS measurements.

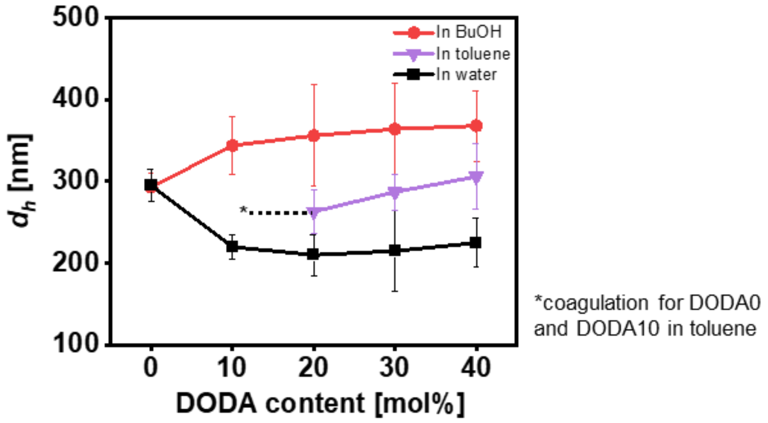

Figure S27. Hydrodynamic diameters of nanogels from DODA0 to DODA40 in various solvents demonstrate an increasing trend in swelling capacity in the order of water, toluene, and 1-butanol.

28. Solubility of non-crosslinked amphiphilic copolymers

To determine the solubility of the non-crosslinked copolymers, 50 mg of each polymer (as prepared in section **11. Preparation of non-crosslinked amphiphilic copolymers**) were added to a 10 mL falcon tube with a stirring bar. Then 1 mL of the respective solvent was added. The tube was sealed with aluminum foil and a cap to avoid evaporation of solvent and the mixture was stirred for one day before centrifugation at 5000 rpm/min for 10 min. Then supernatant was carefully separated from the sediment, dried under vacuum, and weighed to give the mass of polymer dissolved in 1 mL solvent.

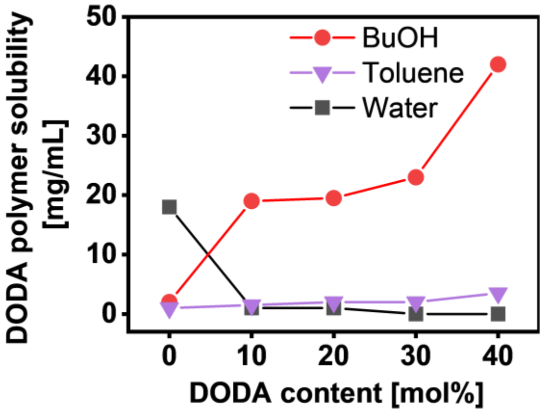

Figure S28. The solubility of non-crosslinked copolymers from DODA0 to DODA40 in various solvents suggests an increasing trend in swelling capacity of the corresponding nanogels in the order of water, toluene, and 1-butanol.

29. Calculation of Flory-Huggins parameters between nanogel network and solvents

Overview of the Flory-Huggins parameters calculation protocol

The Flory-Huggins interaction parameters (FHP) between the nanogels' polymer network and different solvents (i.e., water, different oils) were calculated. The Flory-Huggins parameter (FHP) values serve as indicators of solvent quality for polymers: a value of 0.5 or less suggests that the solvent is a good solvent for the polymer, whereas a value greater than 0.5 implies that the solvent is a poor solvent for the polymer.[6] Classical calculations of Flory-Huggins interaction parameters only involve contributions from the total solubility parameters (δ_T). However, this does not take into consideration the influence of dispersion forces and hydrogen bonding. To include these contributions, we calculated the individual solubility parameters of of network polymer, water, and oil and translated these into the FHP using α as correction constant[6-7] according to equation (6)

(6) $$\chi_{12} = \alpha \frac{V_1}{RT}\left(\left(\delta_{1,\mathrm{D}} - \delta_{2,\mathrm{D}}\right)^2 + 0.25\left(\delta_{1,\mathrm{P}} - \delta_{2,\mathrm{P}}\right)^2 + 0.25\left(\delta_{1,\mathrm{H}} - \delta_{2,H}\right)^2\right)$$

Here, *1* stands for the solvent (water or oil), and *2* stands for the network polymer. α is the correction constant, which, according to the literature[6-7], is used as 0.6 for polymer with oils and 0.3 for polymer with water. V_1 is the molar volume of solvent, R is the gas constant, 8.314 J/(mol·K), T is the temperature (in our case T = 293.15 K). $\delta_{1,D}$, $\delta_{1,P}$ and $\delta_{1,H}$ are the Hansen solubility parameters of the solvent, meaning dispersion solubility parameter, polar solubility parameter and hydrogen bonding solubility parameter, respectively. Similarly, $\delta_{2,D}$, $\delta_{2,P}$, and $\delta_{2,H}$ are the corresponding to Hansen solubility parameters of the network polymer.

The molar volume and Hansen solubility parameters of solvents ($\delta_{1,D}$, $\delta_{1,P}$ and $\delta_{1,H}$) can be found in the handbook.[11]

In contrast, the Hansen solubility parameters of the network polymers ($\delta_{2,D}$, $\delta_{2,P}$, and $\delta_{2,H}$) were calculated by the group contribution method developed by van Krevelen and Hoftyzer[12]. In this method, the following equations are valid for a given molecule:

(7) $$\delta_{\mathrm{D}} = \frac{\Sigma F_{\mathrm{d}i}}{V}$$

(8) $$\delta_P = \frac{\sqrt{\Sigma F_{\mathrm{p}i}^2}}{V}$$

(9) $$\delta_H = \sqrt{\frac{\Sigma E_{\mathrm{h}i}}{V}}$$

Here, i denotes the index of the structural groups, F_d is the dispersion component of the molar attraction function, F_p is the polar component of the molar attraction function, and E_h is the contribution of hydrogen bonding forces to the cohesive energy. The values of F_{di} , F_{pi}^2 , E_{hi} for

each structural group i can be found in the literature[12]. Their sums are denoted as $F_D = \sum F_{di}$, $F_P^2 = \sum F_{pi}^2$, $E_H = \sum E_{hi}$. For all, V is the molar volume, which is calculated by Fedors' method[13].

While these calculations are straightforward for small molecules, the network is composed of a random copolymer from two different monomers, i.e., poly(2-hydroxypropylmethacrylamide-*co*-dodecylmethacrylamide) P(HPMA-*co*-DODMA). Thus, the different energy contributions need to be calculated for each type of repeating unit, i.e., $F_{D,HPMA}$, $F_{D,DODMA}$, $F_{P,HPMA}$, $F_{P,DODMA}$, $E_{H,HPMA}$ and $E_{H,DODMA}$. To account for the random copolymer structure of the network, we need to consider the molar fractions of the different monomers. The overall number of repeating units in each chain does not influence the Hansen solubility parameters and can be neglected. Thus, for P(HPMA-*co*-DODMA), the molar fraction of HPMA in the copolymer is represented by f_{HPMA} and the molar fraction of DODMA is represented by f_{DODMA}. Similarly, the molar volume of HPMA is denoted as $V_{m,HPMA}$, while the molar volume of DODMA is denoted as $V_{m,DODMA}$, respectively. Taking these considerations into account, the three components of the Hansen solubility parameters for the network copolymers can be calculated by:

(10) $$\delta_\mathrm{D} = \frac{f_{HPMA}F_{D,HPMA}+f_{DODMA}F_{D,DODMA}}{f_{HPMA}V_{m,HPMA}+f_{DODMA}V_{m,DODMA}}$$

(11) $$\delta_P = \frac{\sqrt{f_{HPMA}^2F_{P,HPMA}^2+f_{DODMA}^2F_{P,HPMA}^2}}{f_{HPMA}V_{m,HPMA}+f_{DODMA}V_{m,DODMA}}$$

(12) $$\delta_H = \sqrt{\frac{f_{HPMA}E_{H,HPMA}+f_{DODMA}E_{H,DODMA}}{f_{HPMA}V_{m,HPMA}+f_{DODMA}V_{m,DODMA}}}$$

The detailed calculation of F_D, F_P^2, E_H and V_m for one repeating unit of poly(2-hydroxypropylmethacrylamide) (PHPMA) is summarized in Table S6 and Table S7. The detailed calculation of F_D, F_P^2, E_H and V_m for one repeating unit of poly(dodecylmethacrylamide) (PDODMA) is summarized in Table S8 and Table S9.

Calculation of F_D, F_P^2, E_H and V_m for poly(2-hydroxypropylmethacrylamide) (PHPMA)

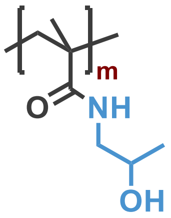

Table S6. Calculation of F_D, F_P^2, and E_H for PHPMA repeating unit.

structural group	number of groups	F_{di} [(J/cm^3)$^{1/2}$·mol^{-1}]	F_{pi}^2 [(J/cm^3)·mol^{-2}]	E_{hi} [J·mol^{-1}]
$-CH_3$	2	2×420	0	0
$-CH_2-$	2	2×270	0	0
>CH−	1	80	0	0
>C<	1	-70	0	0
−C=O−	1	290	770^2	2,000
−NH−	1	160	210^2	3,100
−OH	1	210	500^2	20,000
	in total:	F_D = 2,050	F_P^2 = 887,000	E_H = 25,100

Table S7. Calculation of V_m for PHPMA repeating unit.

structural group	number of groups	V_m [cm^3·mol^{-1}]
monovalent		
$-CH_3$	1	2×33.5
−OH	1	10.0
bivalent		
$-CH_2-$	2	2×16.1
−CONH−	1	9.5
trivalent		
>CH−	1	-1.0
tetravalent		
>C<	1	-19.2
	in total:	98.5

Calculation of F_D, F_P^2, E_H and V_m for poly(dodecylmethacrylamide) (PDODMA)

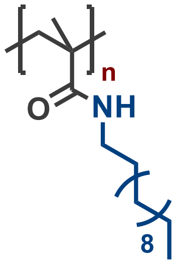

Table S8. Calculation of F_D, F_P^2, and E_H for PDODMA repeating unit.

structural group	number of groups	F_{di} [(J/cm³)$^{1/2}$·mol^{-1}]	F_{pi}^2 [(J/cm³)·mol^{-2}]	E_{hi} [J·mol^{-1}]
$-CH_3$	2	2×420	0	0
$-CH_2-$	12	12×270	0	0
>C<	1	-70	0	0
−C=O−	1	290	770^2	2,000
−NH−	1	160	210^2	3,100
	in total:	F_D = 4,460	F_P^2 = 637,000	E_H = 5,100

Table S9. Calculation of V_m for PDODMA repeating unit.

structural group	number of groups	V_m [cm³·mol^{-1}]
monovalent		
−CH3	2	2×33.5
bivalent		
$-CH_2-$	12	12×16.1
−CONH−	1	9.5
tetravalent		
>C<	1	-19.2
	in total:	250.5

Hansen solubility parameters of copolymers and solvents

Finally, based on the equations (10 – 12), the Hansen solubility parameters of DODA0 – DODA40 are calculated and summarized in Table S10.

Table S10. Hansen solubility parameters of DODA0 – DODA40.

nanogels	δ_D	δ_P	δ_H
DODA0	20.80	9.60	16.00
DODA10	20.15	7.49	14.25
DODA20	19.64	5.97	12.79
DODA30	19.24	4.87	11.51
DODA40	18.92	4.07	10.36

The Hansen solubility parameters and molar volume of the solvents used in this work according to the literature[11] are summarized in Table S11.

Table S11. Hansen solubility parameters and molar volume of the solvents used in this work.[11]

solvents	δ_D	δ_P	δ_H	V_m [$cm^3 \cdot mol^{-1}$]
H_2O	15.50	16.00	42.30	18.0
1-butanol	16.00	5.70	15.80	91.5
1-decanol	17.50	2.60	10.00	191.8
DCM	18.20	6.30	6.10	63.9
toluene	18.00	1.40	2.00	106.8
cyclohexane	16.80	0	0.20	108.7

30. Influence of temperature on the emulsion phase diagram: toluene/water

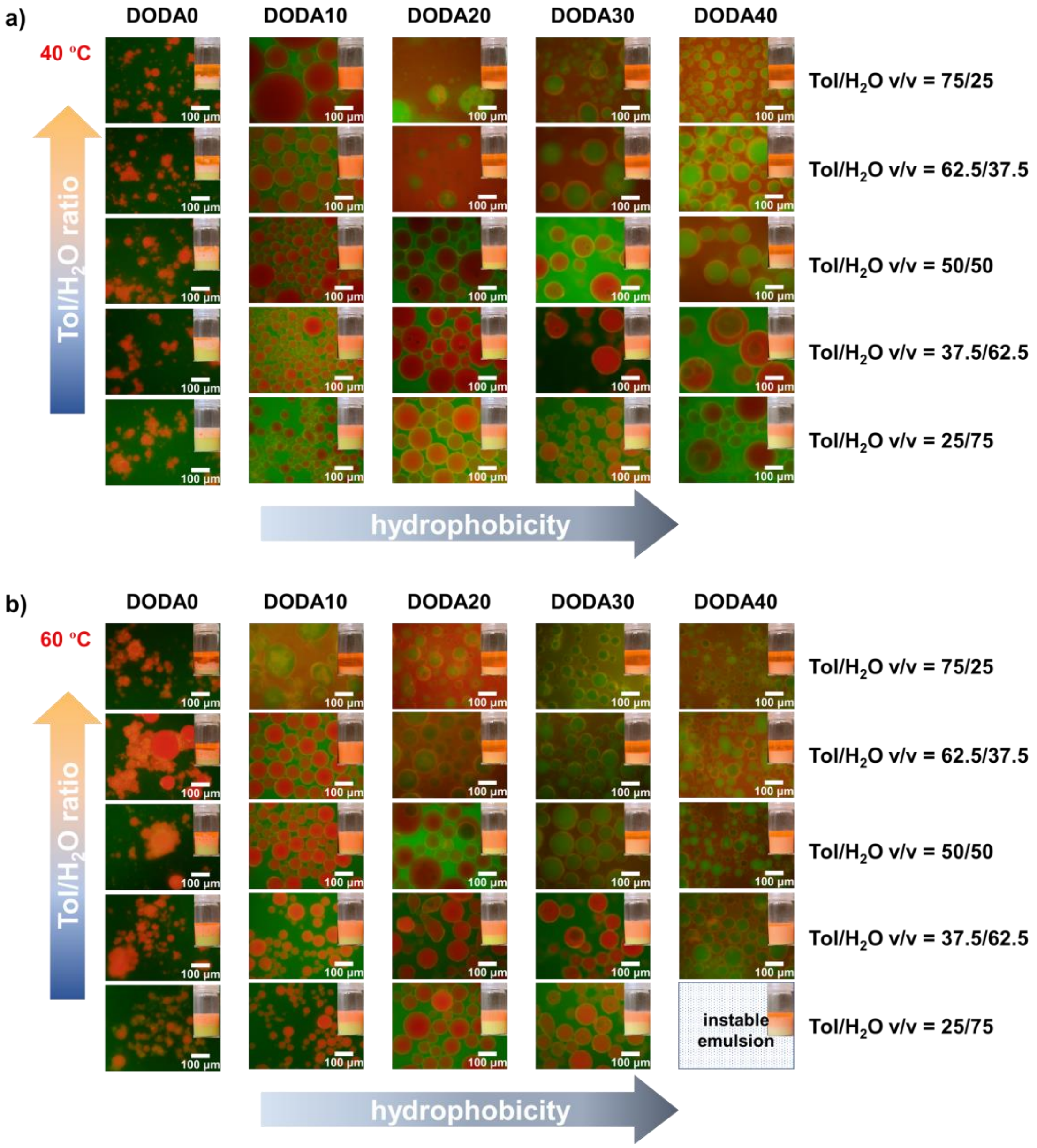

Figure S29. Fluorescence microscopy images and optical photographs of the vials containing the emulsions for the toluene/water system at different emulsification temperatures. Dependence on nanogel hydrophobicity is examined at emulsifying the oil/water system at (a) 40°C and (b) 60°C. Images are taken after cooling to room temperature (20 °C).

31. Examination of temperature-dependent nanogel swelling for DODA40 and DODA30.

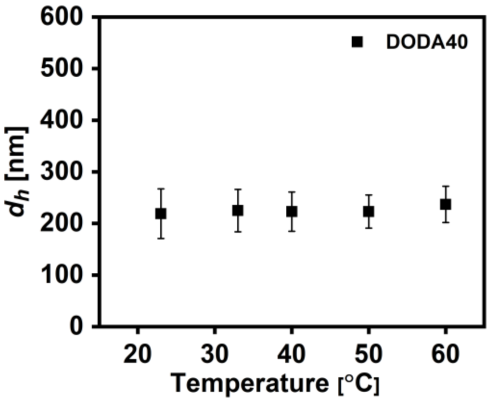

Figure S30. The hydrodynamic diameter of DODA40 nanogels in water shows no temperature responsiveness.

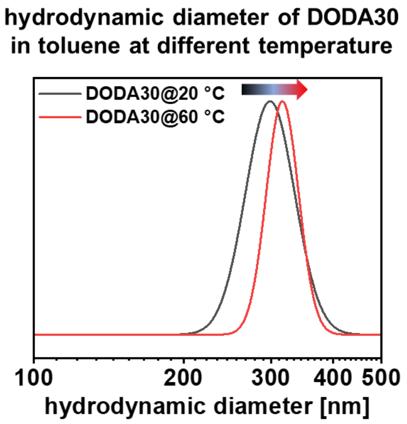

Figure S31. Hydrodynamic diameters (d_h) of DODA30 in toluene at different temperatures. The d_h shows an increase with the temperature increase from 20 ºC to 60 ºC. This increase of swelling of nanogels indicates an increased affinity of DODA30 nanogel to toluene at 60 ºC.

32. Influence of temperature on the emulsion phase diagram: CyH/water

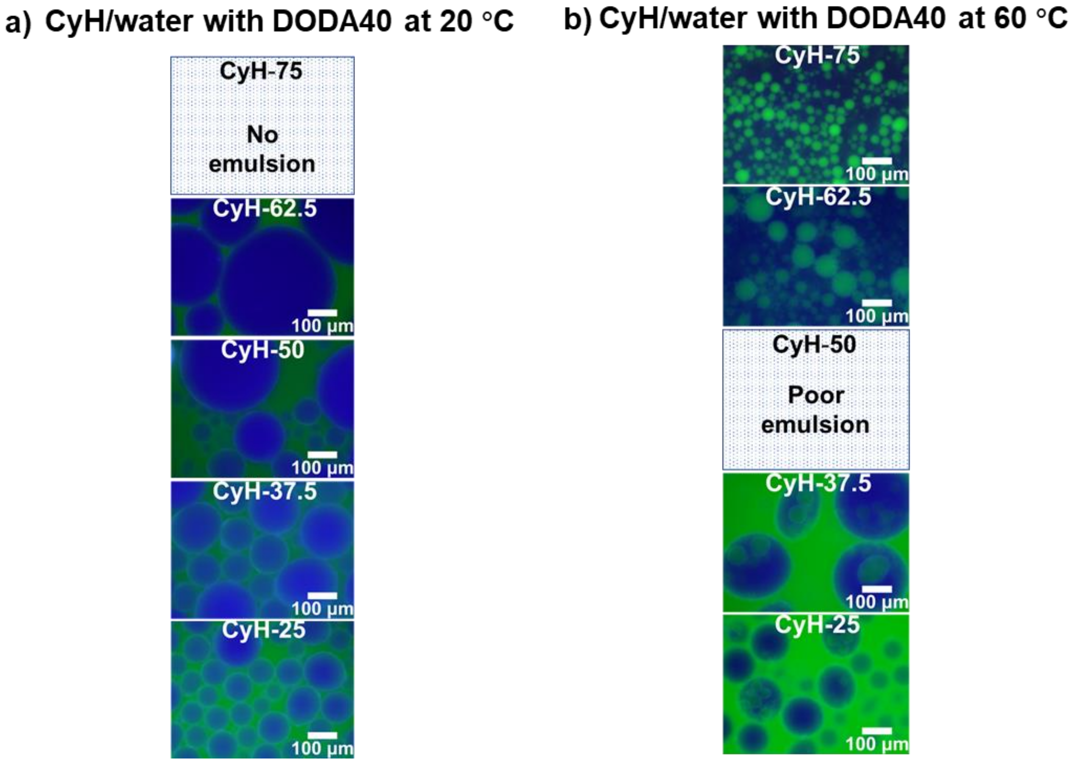

Figure S32. Fluorescence microscopy images of various volume ratios of cyclohexane/water emulsions stabilized with DODA40 nanogels at different emulsification temperatures: (a) 20°C and (b) 60°C. Images are taken after cooling to room temperature (20 °C).

Note that at 40 °C, no emulsions were formed over the whole range of CyH/water ratios. At this temperature, it is assumed that ANGs of intermediate amphiphilicity equilibrate at the middle of the CyH/water interface. This is supported by the phase inversion from O/W at 20 °C to W/O at 60 °C and resembles our observations for the DCM/water system, where poor emulsion stability for DODA10 was correlated to a slow interfacial adsorption.

33. Influence of nanogel hydrophobicity on ROS/water emulsion at 60 °C

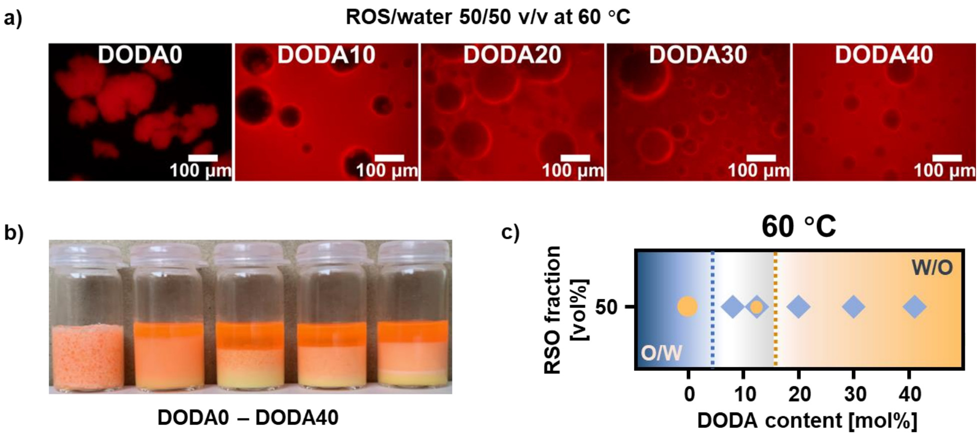

Figure S33. Examination of the rapeseed oil (ROS)/water emulsions stabilized with different nanogels at a temperature of 60 °C. (a) Fluorescence microscopy images. (b) Optical photographs of the vials containing the emulsions. (c) Phase diagram for the 50/50 ROS/water system at 60 °C.

References

(1) Eberhardt, M.; Mruk, R.; Zentel, R.; Théato, P. Synthesis of pentafluorophenyl(meth)acrylate polymers: New precursor polymers for the synthesis of multifunctional materials. *Eur. Polym. J.* **2005**, *41* (7), 1569-1575.

(2) Gruber, A.; Navarro, L.; Klinger, D. Dual-reactive nanogels for orthogonal functionalization of hydrophilic shell and amphiphilic network. *Soft Matter* **2022**, *18* (14), 2858-2871.

(3) Han, K.; Tiwari, R.; Heuser, T.; Walther, A. Simple Platform Method for the Synthesis of Densely Functionalized Microgels by Modification of Active Ester Latex Particles. *Macromol. Rapid Commun.* **2016**, *37* (16), 1323-1330.

(4) Gruber, A.; Isik, D.; Fontanezi, B. B.; Bottcher, C.; Schafer-Korting, M.; Klinger, D. A versatile synthetic platform for amphiphilic nanogels with tunable hydrophobicity. *Polym. Chem.* **2018**, *9* (47), 5553.

(5) Eberhardt, M.; Théato, P. RAFT polymerization of pentafluorophenyl methacrylate: preparation of reactive linear diblock copolymers. *Macromol. Rapid Commun.* **2005**, *26* (18), 1488-1493.

(6) Deshmukh, O. S.; Maestro, A.; Duits, M. H. G.; van den Ende, D.; Stuart, M. C.; Mugele, F. Equation of state and adsorption dynamics of soft microgel particles at an air-water interface. *Soft Matter* **2014**, *10* (36), 7045-7050.

(7) Li, Z. F.; Geisel, K.; Richtering, W.; Ngai, T. Poly(N-isopropylacrylamide) microgels at the oil-water interface: adsorption kinetics. *Soft Matter* **2013**, *9* (41), 9939-9946.

(8) Cayre, O. J.; Paunov, V. N. Fabrication of microlens arrays by gel trapping of self-assembled particle monolayers at the decane–water interface. *J. Mater. Chem.* **2004**, *14* (22), 3300-3302.

(9) Demond, A. H.; Lindner, A. S. Estimation of interfacial tension between organic liquids and water. *Environ. Sci. Technol.* **1993**, *27* (12), 2318-2331.

(10) Shahid, M. Z.; Usman, M. R.; Akram, M. S.; Khawaja, S. Y.; Afzal, W. Initial Interfacial Tension for Various Organic-Water Systems and Study of the Effect of Solute Concentration and Temperature. *J. Chem. Eng. Data* **2017**, *62* (4), 1198-1203.
(11) Hansen, C. M. *Hansen solubility parameters: a user's handbook*; CRC press, 2007.
(12) Van Krevelen, D. W.; Te Nijenhuis, K. *Properties of polymers: their correlation with chemical structure; their numerical estimation and prediction from additive group contributions*; Elsevier, 2009.
(13) Fedors, R. F. A method for estimating both the solubility parameters and molar volumes of liquids. *Polym. Eng. Sci.* **1974**, *14* (2), 147-154.
